# MYC and MET cooperatively drive hepatocellular carcinoma with distinct molecular traits and vulnerabilities

**DOI:** 10.1038/s41419-022-05411-6

**Published:** 2022-11-24

**Authors:** Celia Sequera, Margherita Grattarola, Agnes Holczbauer, Rosanna Dono, Stefania Pizzimenti, Giuseppina Barrera, Kirk J. Wangensteen, Flavio Maina

**Affiliations:** 1 Aix-Marseille Univ, CNRS, Developmental Biology Institute of Marseille (IBDM), Turing Center for Living Systems, Parc Scientifique de Luminy, Marseille, France; 2 Department of Clinical and Biological Science, University of Turin, 10125 Turin, Italy; 3 Division of Gastroenterology, Department of Medicine, Mayo Clinic, Rochester, NY USA

**Keywords:** Cancer models, Liver cancer

## Abstract

Enhanced activation of the transcription factor MYC and of the receptor tyrosine kinase MET are among the events frequently occurring in hepatocellular carcinoma (HCC). Both genes individually act as drivers of liver cancer initiation and progression. However, their concomitant alteration in HCC has not been explored, nor functionally documented. Here, we analysed databases of five independent human HCC cohorts and found a subset of patients with high levels of *MYC* and *MET* (*MYC*^*high*^*/MET*^*high*^) characterised by poor prognosis. This clinical observation drove us to explore the functionality of MYC and MET co-occurrence in vivo, combining hydrodynamic tail vein injection for *MYC* expression in the *R26*^*stopMet*^ genetic setting, in which wild-type MET levels are enhanced following the genetic deletion of a stop cassette. Results showed that increased *MYC* and *MET* expression in hepatocytes is sufficient to induce liver tumorigenesis even in the absence of pre-existing injuries associated with a chronic disease state. Intriguingly, ectopic *MYC* in *MET* tumours increases expression of the *Mki67* proliferation marker, and switches them into loss of *Afp, Spp1, Gpc3, Epcam* accompanied by an increase in *Hgma1, Vim*, and Hep-Par1 levels. We additionally found a switch in the expression of specific immune checkpoints, with an increase in the *Ctla-4* and *Lag3* lymphocyte co-inhibitory responses, and in the *Icosl* co-stimulatory responses of tumour cells. We provide in vitro evidence on the vulnerability of some human HCC cell lines to combined MYC and MET targeting, which are otherwise resistant to single inhibition. Mechanistically, combined blockage of MYC and MET converts a partial cytostatic effect, triggered by individual blockage of MYC or MET, into a cytotoxic effect. Together, these findings highlight a subgroup of HCC characterised by *MYC*^*high*^*/MET*^*high*^, and document functional cooperativity between MYC and MET in liver tumorigenesis. Thus, the *MYC-R26*^*Met*^ model is a relevant setting for HCC biology, patient classification and treatment.

## Introduction

Hepatocellular carcinoma (HCC) is among the most aggressive and heterogeneous types of cancer, with an increasing incidence, and few treatment options [1–3]. In a vast majority of cases, HCC originates in the setting of fibrosis and cirrhosis due to chronic viral hepatitis (HBV and HCV) infection, and alcoholic or non-alcoholic liver disease [4, 5]. Nevertheless, a growing number of HCC cases in patients arises also in the absence of cirrhosis. This has been supported by several mouse models and elegant genetic screens, illustrating how forced alterations of clinically relevant genes in normal hepatocytes, in the absence of preceding liver damages, are sufficient to trigger tumour formation [6–9]. These “inside-out” models of HCC are particularly useful to functionally test genetic combinations driving the HCC programme in the absence of multiple, secondary effects associated with a chronic disease state, such as persistent regenerative processes, cirrhosis, hepatitis infections or drastic metabolic alterations linked to alcohol and obesity, as is the case in the “outside-in” models [10]. Moreover, in the “outside-in” models, the molecular processes at the roots of hepatocarcinogenesis initiation and progression do not always reflect those occurring in humans. Thus, assessing co-occurring alterations based on clinical data with “inside-out” models can dissect the functionality and cooperativity of liver cancer drivers [10].

The most effective current HCC therapy, which has only a ~25% tumour response rate, depends on treating the tumour microenvironment by blocking PD-L1 to activate immune cells (Atezolizumab) plus inhibiting tumour vascularity (Bevacizumab) [11]. These drugs showed improvement over existing therapy with receptor tyrosine kinase inhibitors. Additional tumour-intrinsic targets are needed to further improve outcomes without increasing the toxicity profile. The outcomes could be tremendously beneficial, even if only for a subfraction of patients.

*MYC* and *MET* genes drive HCC pathology. The *MYC* gene is amplified in several human cancers and overexpressed in up to 70% of viral and alcohol-related HCC [12, 13]. The amplification of the *MYC* locus is one of the earliest events in HCC formation [14]. In mouse models, ectopic expression of *MYC* in combination with other oncogenes initiates and drives HCC [6]. Genetic modelling of HCC has indicated that blocking MYC leads to tumour regression, suggesting that HCC can become MYC oncogene-addicted [15, 16]. Concerning the receptor tyrosine kinase (RTK) MET, although mutations are rare in HCC and they predominantly occur in paediatric HCC [17, 18], it is activated in close to 50% of cases [19], participates in tumour-stroma crosstalk [20], and correlates with poor prognosis [21, 22]. Transgenic mouse models with oncogenic HGF/MET develop liver tumours [23]. Overall, the evidence implicating MET in HCC is sufficiently strong to have warranted several clinical trials of MET inhibitors [24]. Using a unique genetic setting in which expression of wild-type MET can be slightly enhanced above its endogenous level in a tissue-specific manner (*R26*^*stopMet*^ mice) [25–28], we have documented how liver-enhanced MET leads progressively to tumorigenesis with age, reaching approximately 80% frequency (*Alb-R26*^*Met*^ mice) [29, 30]. The *Alb-R26*^*Met*^ can be considered as a predisposition model, as the slightly enhanced MET levels leave hepatocytes vulnerable to the emergence of molecular events that trigger preneoplastic lesions and progression towards HCC. This is exemplified by a transposon mutagenesis screen we have performed in *Alb-R26*^*Met*^ mice, which illustrated an extraordinary vulnerability of the liver to various additional alterations leading to tumorigenesis [30]. The *Alb-R26*^*Met*^ setting as an “open” predisposition model recapitulates several features of HCC patients: the molecular heterogeneity, the primary resistance to drugs used in the clinic, the temporal heterogeneity of tumour onset [29], and the enrichment in genes both overexpressed and hypermethylated in gene body CpG islands occurring in 56% of proliferative-progenitor HCC patients [31]. Among these genes, we recently documented *ADAMTSL5*, not previously linked to cancer, which could be a new biomarker and target for HCC [32]. The *Alb-R26*^*Met*^ mice have been instrumental in showing how C3G (RapGEF1) ensures the full activation of the HGF/MET signalling pathway in HCC [33]. Moreover, they have been used as a relevant genetic setting to show how enhanced MET levels impact hepatic glucose homoeostasis and attenuate insulin-mediated signalling [34]. Despite the relevance of MYC and MET in HCC, neither their co-occurring alteration nor their functional cooperativity has been explored.

Here, we report that a subgroup of HCCs co-expresses *MYC* and *MET* at high levels. This clinical observation drove us to explore the functionality of MYC and MET co-occurrence in vivo, combining hydrodynamic tail vein injection for *MYC* expression with the *R26*^*stopMet*^ genetic setting, in which wild-type MET levels are enhanced following the deletion of the stop cassette by Cre recombinase. We demonstrated that *MYC* and *MET* cooperate to trigger liver tumorigenesis in vivo, modelling the subgroup of HCC patients with high levels of both genes. Interestingly, *MYC* expression led to a switch in the expression of a set of markers of HCC and of immune checkpoints. Finally, we provide evidence on the in vitro vulnerability of some human HCC cell lines to combined MYC and MET targeting, otherwise resistant or only moderately responding to single inhibition. Mechanistically, this combinatorial inhibition converts a mild cytostatic effect following a single MYC or MET blockage into a drastic cytotoxic effect when both signals are targeted.

## Results

### A subset of HCCs co-expresses high levels of MYC and MET

**Fig. 1:**
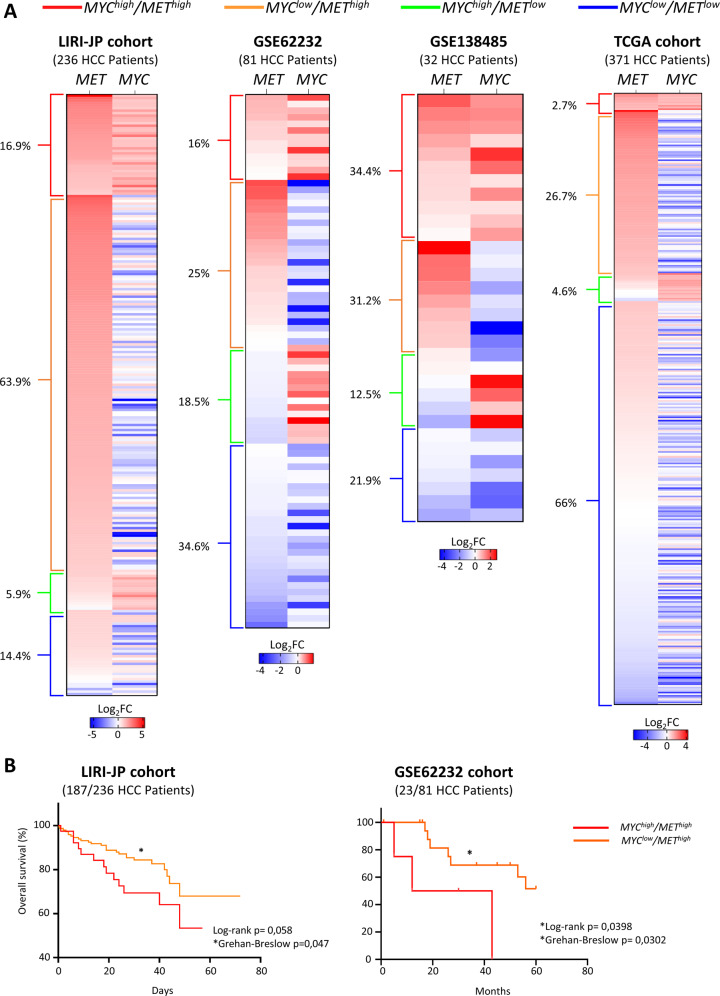
High expression levels of *MYC* and *MET* co-occur in a subset of HCC patients. **A** Heatmap reporting four different cohorts (LIRI-JP, TGGA-LIHC, GSE62232, and GSE138485) with HCC patients organised according to the expression levels of *MYC* and *MET*, and subdivided into four subgroups: *MYC*^*high*^*/MET*^*high*^, *MYC*^*low*^*/MET*^*high*^, *MYC*^*high*^*/MET*^*low*^, and *MYC*^*low*^*/MET*^*low*^. The percentage of patients in each subgroup is indicated on the left of each heatmap. **B** Kaplan–Meier curve showing the overall survival of LIRI-JP (left) and GSE62232 (right) patients with *MYC*^*high*^*/MET*^*high*^ versus *MYC*^*low*^*/MET*^*high*^. Note that for the GSE62232 cohort, survival information was available for only four patients of the *MYC*^*high*^*/MET*^*high*^ group. Statistical analysis was performed with Grehan–Breslow.

Previous studies have shown that MYC overexpression throughout development can drive HCC formation [35], but an expression of MYC by hydrodynamic tail vein injection in C57BL6 only results in tumours in combination with other oncogenic drivers [6]. We explored whether high expression levels of MYC and MET co-occur in five different human HCC cohorts. These analyses revealed 40 out of 236 (16.9%) *MYC*^*high*^*/MET*^*high*^ HCC patients in the LIRI-JP cohort (Fig. 1A). In two other independent cohorts of HCC patients, 13 out of 81 (16%; from GSE62232) and 11 out of 32 (34.4%; from GSE138485) were *MYC*^*high*^*/MET*^*high*^ (Fig. 1A). We analysed the HCC French cohort (LICA-FR) and found 92 out of 161 (57.1%) were *MYC*^*high*^*/MET*^*high*^ (Fig. S1). A smaller proportion of *MYC*^*high*^*/MET*^*high*^ HCC patients was found in the TCGA cohort (10 out of 371; 2.7%; Fig. 1A). Thus, approximately 18.8% of all HCC analysed patients (119/881 total cases) are *MYC*^*high*^*/MET*^*high*^.

We analysed the clinical features of the subset of patients with *MYC*^*high*^*/MET*^*high*^ HCCs. Patients had shorter overall survival compared with *MYC*^*low*^*/MET*^*high*^ (Fig. 1B). *MYC*^*high*^*/MET*^*high*^ HCC patients had no molecular features, aetiology, or mutations as compared to *MYC*^*low*^*/MET*^*high*^ HCC patients. The only intriguing point might be the different underlying aetiology of liver disease in the distinct cohorts, with the Japanese (LIRI-JP) cohort predominantly viral-related HCC, whereas the GSE138485 mostly non-HBV, the French cohort (LICA-FR) secondary to alcohol and adiposity, the US cohort (TCGA) and GSE62232 with a mixed aetiology.

### Concomitant upregulation of MYC and MET in a subset of hepatocytes triggers tumorigenesis in mice

**Fig. 2:**
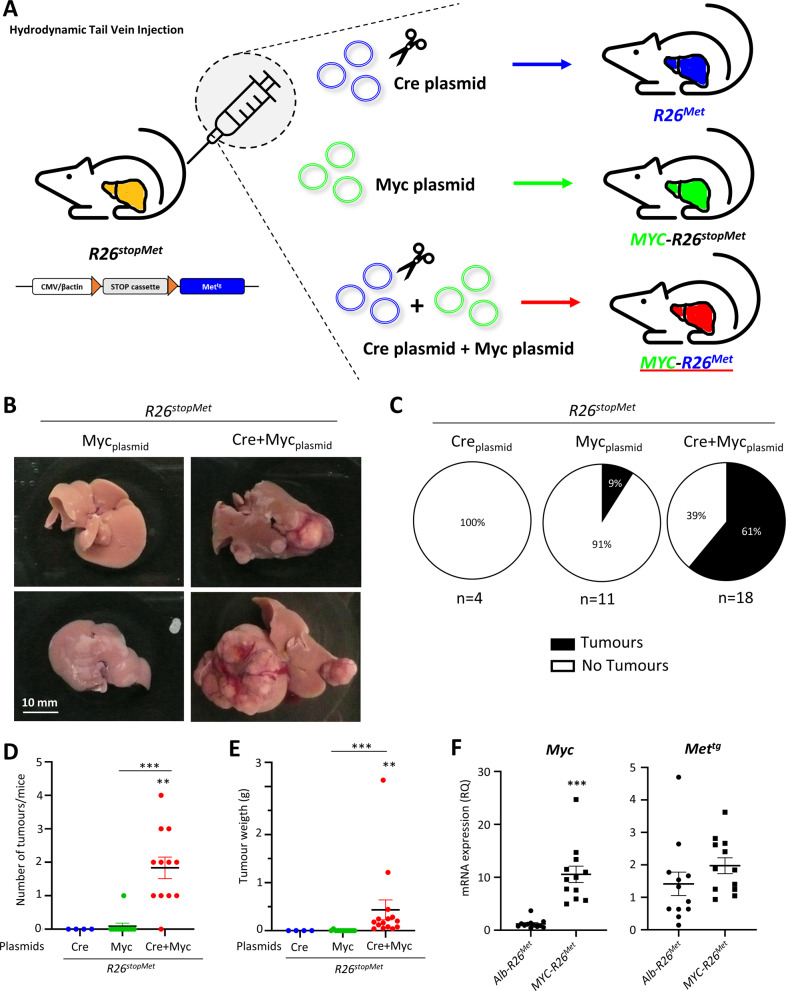
Concomitant upregulation of MYC and MET in a subset of hepatocytes triggers tumorigenesis in mice. **A** Scheme illustrating the protocol of hydrodynamic tail vein injection used in *R26*^*stopMet*^ mice with *Cre*, *Myc*, or *Cre* + *Myc* plasmids. **B** Liver representative photographs of hydrodynamically injected *R26*^*stopMet*^ mice with *Myc* or *Cre* + *Myc* plasmids. **C** Pie charts representing the percentage of *R26*^*stopMet*^ mice with or without tumours (black and white, respectively) after hydrodynamic injection of *Cre*, *Myc*, or *Cre* + *Myc* plasmids. **D**, **E** Dot plots representing the number of tumours per mouse (**D**) and the tumour weight (**E**) in *R26*^*stopMet*^ mice hydrodynamically injected with *Cre*, *Myc*, or *Cre* + *Myc* plasmids. **F** Dot plots reporting the mRNA expression levels of *Myc* and *Met*^*tg*^ analysed by RT-qPCR in dissected *Alb-R26*^*Met*^ and *Myc-R26*^*Met*^ tumours, normalised using the *Gapdh* housekeeping gene and expressed as RQ. Statistical analyses were performed by Mann–Whitney. *p ≤ 0.05; **p ≤ 0.01; ***p ≤ 0.001.

We next assessed whether the clinical co-existence of high *MYC* and *MET* levels in HCC patients is functionally relevant to drive liver cancer. We reasoned that forced MYC expression in the *R26*^*stopMet*^ genetic setting could be an appropriate system to model this patient subgroup. We performed hydrodynamic tail vein injection of two plasmids for (a) transient expression of the Cre recombinase, to allow MET^tg^ expression by deleting a stop cassette (*Cre* plasmid); (b) transient expression of the Sleeping Beauty transposase to trigger the genomic insertion of the *Myc* transgene (*Myc* plasmid; Fig. 2A). We found that both control groups, with either the *Cre* or the *Myc* plasmid alone, followed up to 24 weeks, did not develop any macroscopic signs of tumorigenesis (Fig. 2B, C). Instead, 11/12 *Myc-R26*^*Met*^ mice (generated by hydrodynamic tail vein injection with both *Cre* and *Myc* plasmids) developed tumours (Fig. 2B–D). Tumour weight ranged between 0.39 and 1.21 g, with an additional, big tumour that reached 3.13 g (Fig. 2E). Reverse transcription-quantitative polymerase chain reaction (RT-qPCR) analysis confirmed upregulation of *Myc* in *Myc-R26*^*Met*^ compared with *Alb-R26*^*Met*^ tumours (Fig. 2F). Similar expression levels of *MET*^*tg*^ were found in both *Myc-R26*^*Met*^ and *Alb-R26*^*Met*^ tumours (Fig. 2F). Collectively, these results show that MYC and MET cooperate to trigger liver tumorigenesis in mice.

### MYC upregulation switches the molecular identity of HCC in MET cancer models

**Fig. 3:**
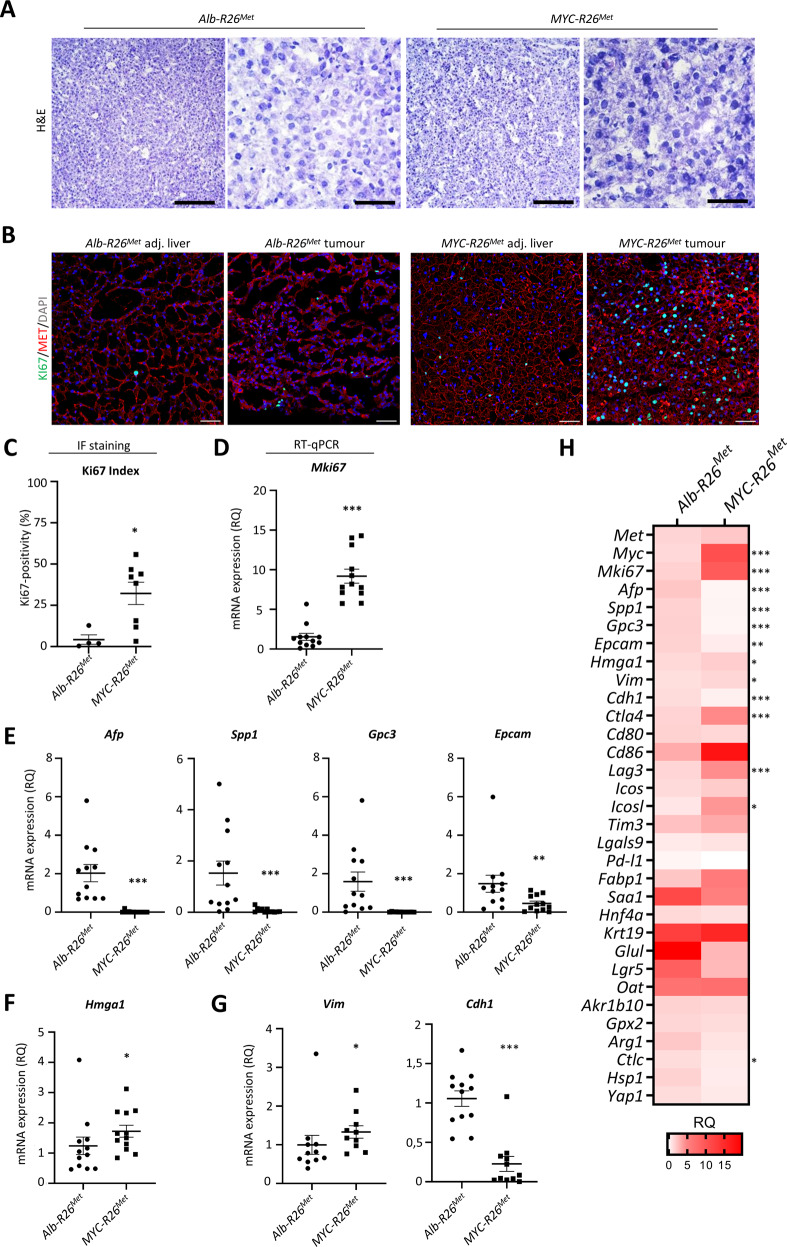
Hepatocellular characteristics of *Myc-R26*^*Met*^ and *Alb-R26*^*Met*^ tumours. **A** Representative haematoxylin and eosin staining of frozen, fixed *Alb-R26*^*Met*^ and *Myc-R26*^*Met*^ tumours and adjacent livers. **B**, **C** Representative images (**B**) and graph with quantifications (**C**) of Ki67 immunofluorescence staining of *Alb-R26*^*Met*^ and *Myc-R26*^*Met*^ tumour sections (scale bar: 50 µm). **D**–**G** mRNA expression levels by RT-qPCR of the proliferation marker *Mki67* (**D**), of HCC markers *Afp*, *Spp1*, *Gpc3*, and *Epcam* (**E**), of the non-histone chromatin protein *Hmga*1 (**F**), and of mesenchymal *Vim* and epithelial *Cdh1* markers (**G**) in *Myc-R26*^*Met*^ versus *Alb-R26*^*Met*^ tumours. **H** Heatmap reporting the RQ differential expression of all genes evaluated by RT-qPCR in this study. Values were normalised with the *Gapdh* housekeeping gene and expressed as RQ, all values relative to *Alb-R26*^*Met*^ tumours. Statistical analyses were performed by Mann–Whitney. *p ≤ 0.05; **p ≤ 0.01; ***p ≤ 0.001.

A review of Haematoxylin/Eosin staining with a pathologist confirmed that the tumours had characteristics of moderately to well-differentiated HCC consisting of polygonal tumour cells arranged in a solid or trabecular pattern (Fig. 3A). Immunofluorescence staining of Ki67 revealed a significantly higher proliferation index in *Myc-R26*^*Met*^ versus *Alb-R26*^*Met*^ tumours (Fig. 3B, C). Results were confirmed by RT-qPCR analysis of *Mki67* mRNA levels, showing a significantly higher proliferation rate in *Myc-R26*^*Met*^ compared with *Alb-R26*^*Met*^ tumours (Fig. 3D).

To further characterise the *Myc-R26*^*Met*^ tumours, we analysed the mRNA levels by RT-qPCR of sets of markers. We found significantly decreased levels of *Afp* (a marker of undifferentiated HCC), *Spp1*, *Gpc3* (markers of early HCC), and *Epcam* (a marker of stemness; Fig. 3E, H), suggesting that *Myc-R26*^*Met*^ tumours might be more differentiated and at more advanced stages than *Alb-R26*^*Met*^ tumours. However, we did not find any differences in the mRNA expression of other markers related to HCC characterisation (*Saa1, Fabp1*), progenitor cells *(Hnf4a, Krt19*), Wnt pathway (*Glul, Lgr5, Oat*), metabolism (*Ark1b10, Gpx2*), and differentiated markers (*Arg1, Ctlc, Hsp1, Yap1*) in *Myc-R26*^*Met*^ versus *Alb-R26*^*Met*^ tumours (Figs. 3H, S2).

Additionally, we examined the role of HMGA1, a non-histone chromatin-related protein that was recently described as a marker overexpressed in MYC-negative triple-negative breast cancer [36]. HMGA1 is a potent oncogene that triggers tumour progression and is related to undifferentiated stem-like phenotypes and aggressiveness [37]. It has been recently shown that HMGA1 is part of a positive feedback loop dependent on MYC to promote stemness and epithelial–mesenchymal transition [38]. We found that *Hgma1* is upregulated in *Myc-R26*^*Met*^ compared with *Alb-R26*^*Met*^ tumours (Fig. 3F, H), consistent with its involvement in MYC regulation as recently reported. Reassuringly, *Eif4e*, another MYC target gene, was also upregulated in *Myc-R26*^*Met*^ tumours (Fig. S2A).

**Fig. 4:**
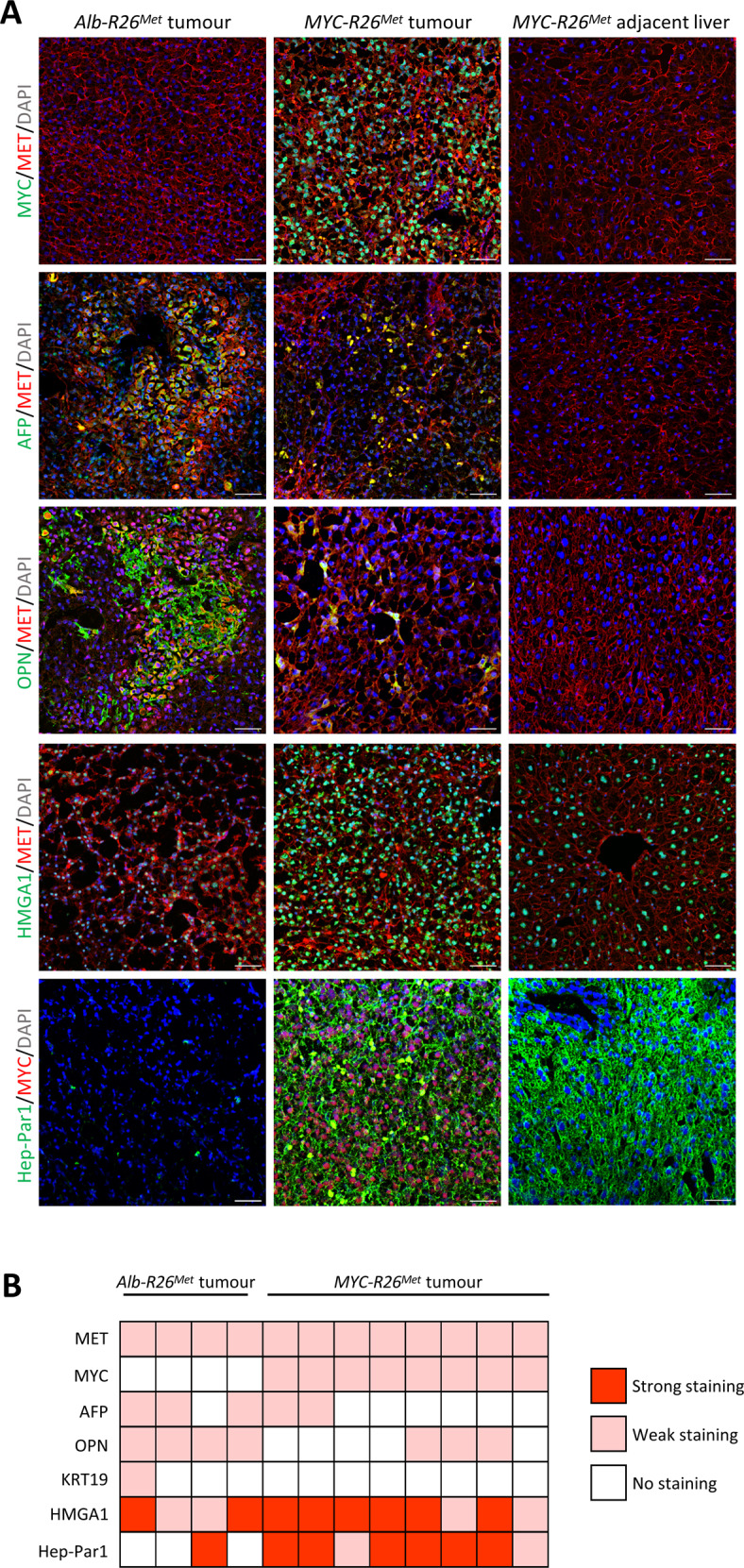
Molecular characteristics of *Myc-R26*^*Met*^ and *Alb-R26*^*Met*^ tumours. **A** Representative images of immunofluorescence staining of *Myc-R26*^*Met*^ and *Alb-R26*^*Met*^ tumour sections (and *Myc-R26*^*Met*^ adjacent livers as controls), documenting the expression of MET, MYC, AFP, OPN, and EPCAM (scale bar: 50 µm). Nuclear staining in DAPI (blue). **B** Heatmap reporting the intensity of staining of immunofluorescence images for proteins reported on the left. Each square represents a distinct tumour sample.

To further characterize the phenotypic switch, we also analysed the mRNA levels of well-described epithelial-to-mesenchymal transition (EMT) markers, in relation to the capability of MYC to promote EMT in solid tumours [39, 40]. We observed an increase of Vimentin (*Vim*) mesenchymal marker and a decrease in E-cadherin (*Cdh1*) epithelial marker in *Myc-R26*^*Met*^ compared with *Alb-R26*^*Met*^ tumours (Fig. 3G, H), indicating a more mesenchymal phenotype for *Myc-R26*^*Met*^, which has been associated with aggressiveness, poor prognosis and resistance to drugs currently used in the clinics [41, 42]. Next, we performed immunofluorescence analysis to further document the molecular switch found in *Myc-R26*^*Met*^ versus *Alb-R26*^*Met*^ tumours. We confirmed MET expression in both *Alb-R26*^*Met*^ and *Myc-R26*^*Met*^ tumours, whereas high MYC levels were restricted to *Myc-R26*^*Met*^ tumours (Fig. 4A, B). Reassuringly, we found a decrease in AFP and OPN and an increase in HMGA1 and Hep-Par1 staining in *Myc-R26*^*Met*^ versus *Alb-R26*^*Met*^ tumours (Fig. 4A, B).

**Fig. 5:**
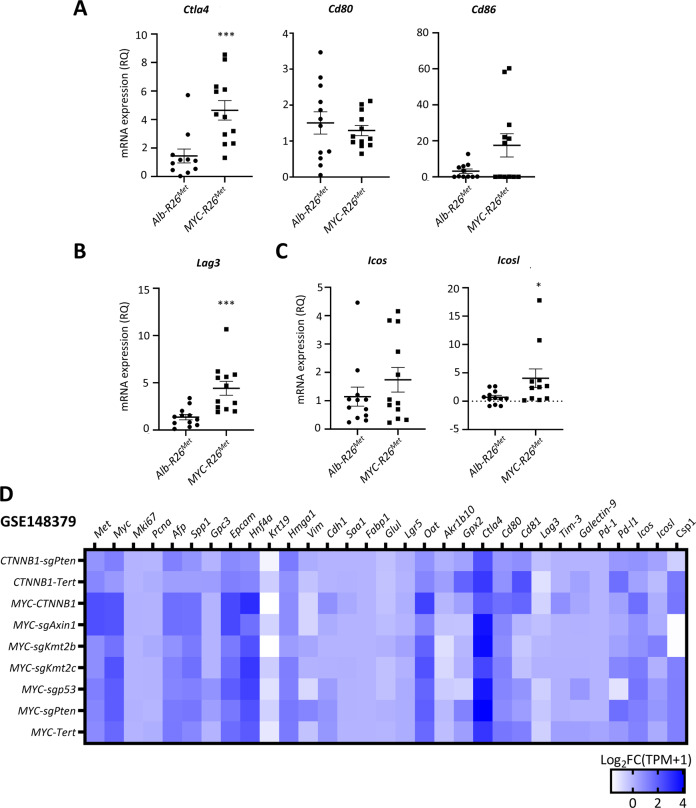
MYC upregulation switches the immune-related molecular identity of HCC in MET cancer models. **A**–**C** Graphs report the mRNA expression levels by RT-qPCR of the immune checkpoints *Ctla4* and its ligands *Cd80* and *Cd81* (**A**), *Lag3* (**B**), *Icos* and its ligand *Icosl* (**C**). **D** Heatmap representing the mRNA levels of genes of interest (indicated on the top) expressed as Log_2_FC (TPM + 1) in different tumours from murine HCC models obtained by hydrodynamically injecting plasmids to overexpress MYC in combination with different oncogenes (indicated on the left), compared with control livers. Results were extracted from a publicly available database with the GEO accession number GSE148379.

Recent studies have described MYC as a remodeler of the immune microenvironment in different solid cancers [43, 44]. We therefore explored by RT-qPCR whether there was a switch in immune checkpoints in *Myc-R26*^*Met*^ versus *Alb-R26*^*Met*^ tumours. Interestingly, we found higher expression of *Ctla4* and *Lag3* in *Myc-R26*^*Met*^ compared with *Alb-R26*^*Met*^ tumours (Fig. 5A, B). The upregulation of *Ctla4* and *Lag3* was rather specific as no significant changes were observed in mRNA levels of *Cd80* and *Cd86* (two CTLA4 ligands present in the antigen-presenting (tumour) cells), *Tim-3/Galectin-9* (*Havcr2*/*Lgals9*), and *Pd-l1* (*Cd274*) immune checkpoints (Figs. 5A, S2E, F). Moreover, we found slightly increased mRNA levels of *Icosl* (expressed by tumour cells), but not of its receptor *Icos* (expressed by Lymphocyte T), in *Myc-R26*^*Met*^ versus *Alb-R26*^*Met*^ tumours, indicating a putative presence of co-stimulatory response (Fig. 5C). We could not detect by IHC CD3-positive cells in tumours of both genotypes (Fig. S3), indicating that differences in the expression of immune checkpoints we detected might be linked to altered crosstalks between immune and cancer cells or to changes in the composition of specific immune cells subtypes.

Finally, we asked whether the switch in gene expression in MET tumours following MYC overexpression occurs in other murine models in which HCC is triggered by hydrodynamic co-injection of plasmids driving expression of MYC in combination with different known oncogenes (GSE148379) [6]. Surprisingly, we found that overexpression of MYC with oncogenes other than MET did not produce a striking change in the expression of genes switched in *Myc-R26*^*Met*^ versus *Alb-R26*^*Met*^ tumours (*Afp, Spp1, Gpc3, Epcam, Mki67*, *Hgma1*, *Csp1* (Hep-Par1)*, Vim*, *Cdh1* markers, and *Ctla4*, *Lag3*, *Icosl* immune checkpoints; Fig. 5D). Together, these findings revealed an intriguing switch in the levels of specific markers when MYC overexpression occurs in the setting of upregulated MET.

### Combinatorial targeting of MYC and MET confers responsiveness in a subset of human HCC cells, otherwise resistant to a single treatment

**Fig. 6:**
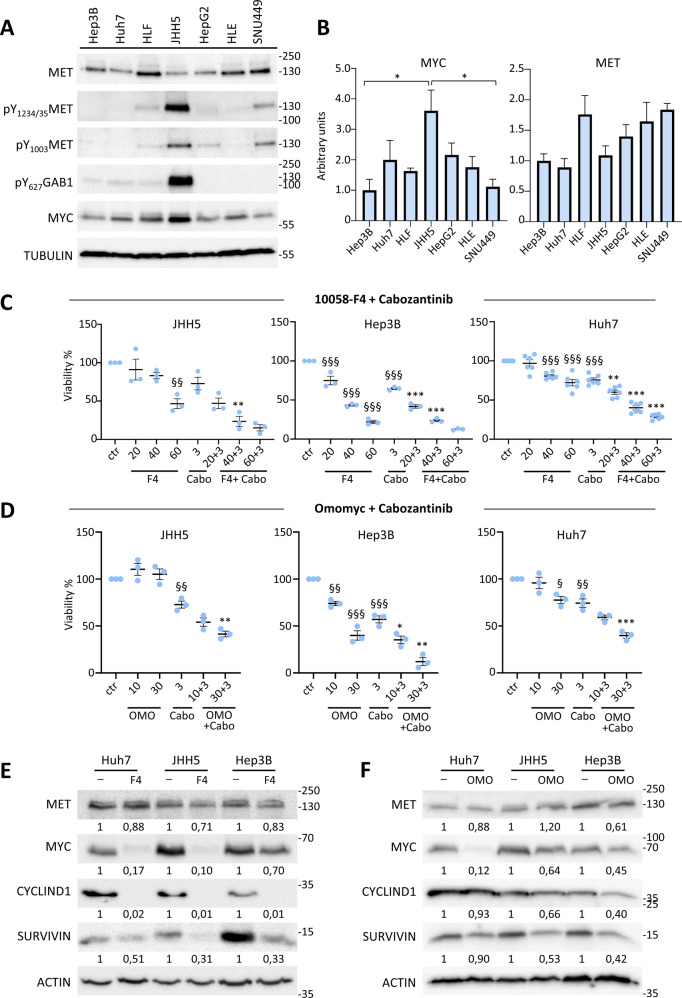
MYC targeting confers responsiveness of a subset of HCC cell lines to cabozantinib treatment. **A** Western blot reporting expression and phosphorylation levels of the indicated proteins in a panel of human HCC cell lines. ACTIN and TUBULIN were used for normalisation (full blots of gels are reported in Figs. S6 and S7). **B** Graphs reporting quantifications of expression of the indicated proteins, based on densitometric analysis by Image J. Measures were normalised using ACTIN or TUBULIN; the quantification was done setting as 1 the expression of the cell line with the lowest amount of protein. *p < 0.05. **C** Graphs reporting cell viability assays performed using 10058-F4 (20, 40, and 60 µM) either alone or with cabozantinib (3 µM) in the indicated human HCC cell lines. With 10058-F4 (40 µM), reduced viability to: 83.24% in JHH5, *p* > 0.05; 43.33% in Hep3B, *p* < 0.0001; 80.68% in Huh7, *p* < 0.001. With cabozantinib, reduced viability to: 72.87% in JHH5, *p* > 0.05; 64.58% in Hep3B, *p* < 0.0001; 75.74% in Huh7, *p* < 0.0001. With 10058-F4 + cabozantinib, reduced viability to: 23.60% in JHH5, *p* < 0.01; 23.83% in Hep3B, *p* < 0.001; 40.15% in Huh7, *p* < 0.0001. **D** Graphs reporting cell viability assays performed using Omomyc (10 and 30 µM) either alone or with cabozantinib (3 µM) in the indicated human HCC cell lines. With Omomyc, reduced viability to: 74.03% and 39.94% in Hep3B cells with 10 and 30 µM, *p* < 0.01 and *p* < 0.0001, respectively. With Omomyc+cabozantinib, reduced viability to: 54.11% and 41.50% in JHH5 cells with 10 and 30 µM, *p* > 0.05 and *p* < 0.01; 35.31% and 12.12% in Hep3B cells with 10 and 30 µM, *p* < 0.05 and *p* < 0.01; 59.13% and 39.84% in Huh7 cells with 10 and 30 µM, *p* > 0.05 and *p* < 0.001). In **C** and **D**, three to six independent experiments were done. **E**, **F** Western blots reporting the expression levels of the indicated proteins in human HCC cells untreated and treated with 10058-F4 (60 µM; **E**) or with Omomyc (30 µM; **F**). Note a consistent downregulation of SURVIVIN, CYCLIN D1, and MYC levels in the analysed cell lines exposed to either Omomyc or 10058-F4. Unchanged or slight downregulation of MET levels was observed following MYC blockage, coherent with the sensitivity of cells to MET inhibition by cabozantinib shown in panels (**C**) and (**D**). Statistical analyses were performed by (one-way) ANOVA. ^§^*p* < 0.05; ^§§^*p* < 0.01; ^§§§^*p* < 0.001; *p < 0.05; **p < 0.01; ***p < 0.001. § indicates 10058-F4 or Omomyc and cabozantinib (cabo) versus controls (ctr); * indicates 10058-F4 + Cabo or Omomyc+Cabo versus respectively 10058-F4 or Omomyc and Cabozantinib.

Based on transcriptomics data, a panel of human liver cancer cell lines has previously been subdivided in three subgroups [45]. The CL1 subgroup corresponds to most differentiated cells, distinct for expression of epithelial and foetal/progenitor markers, whereas the CL3 corresponds to less differentiated cells, with mesenchymal traits, and invasive and stem cell-like markers. The CL2 subgroup corresponds to cells with mixed epithelial–mesenchymal, hepato-specific and stem cell-like features [45]. By analysing data available on https://lccl.zucmanlab.com/hcc/molecularFeatures/rnaExpression?index=1, we found no correlation between expression levels of MYC or MET and CL subgroups (Fig. S4A–C). Moreover, no mutations in *MYC* or *MET* genes are reported in this liver cancer cell panel, except for a *MYC* missense mutation in HCC1.1 and *MET* amplification in MHCC97 cells. No correlation was found between MYC and MET levels, only a non-significant trend for the CL3 subgroup (Pearson: 0.1796; *p* = 0.31; Fig. S3B, C). We, therefore, selected a subset of CL1 (Hep3B, Huh7, JHH5, HepG2) and of CL3 (HLE, HLF, SNU449) human HCC cells to analyse MYC and MET protein levels in cell extracts. We found that cells express slightly different degrees in levels of MYC and MET (Fig. 6A, B), without any evident correlation, as shown by transcriptomic data (Fig. S4). JHH5 cells are characterised by MET phosphorylation and activation of the downstream GAB1 signal, consistent with HGF expression (https://lccl.zucmanlab.com/hcc/molecularFeatures/rnaExpression/HGF?index=1&cid=10207) and autocrine MET activation (Figs. 6A, S5A).

**Fig. 7:**
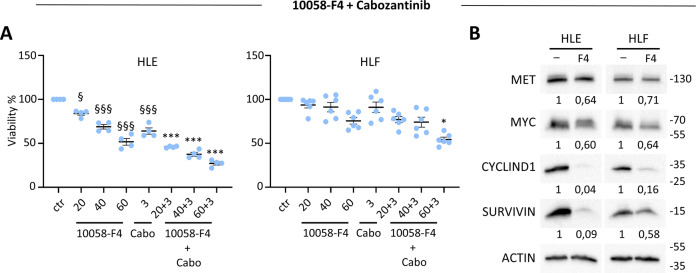
MYC targeting sensitises HCC cell lines belonging to the CL3 subgroup to cabozantinib treatment. **A** Graphs reporting data of cell viability assays performed using 10058-F4 (20, 40, and 60 µM) either alone or with cabozantinib (3 µM) for 72 h in HLE and HLF human HCC cell lines. 10058-F4 (60 µM) reduced viability to: 51.77% in HLE, *p* < 0.0001; 75.62% in HLF, *p* < 0.01. Cabozantinib reduced viability to: 64.01% in HLE, *p* < 0.0001; 91.11% in HLF, *p* > 0.05. 10058-F4 + cabozantinib reduced viability to: 27.20% in HLE, *p* < 0.0001; 54.31% in HLF, *p* < 0.05. **B** Western blots reporting the expression levels of the indicated proteins in human HCC cells untreated and treated with 10058-F4 (60 µM). Note a consistent downregulation of SURVIVIN, CYCLIN D1, and a partial reduction of MYC levels in HLE and HLF exposed to 10058-F4. Slight downregulation of MET levels was observed following MYC blockage, supporting the sensitivity of cells to MET inhibition by cabozantinib shown in panel (**A**). Statistical analyses were performed by (one-way) ANOVA. ^§^*p* < 0.05; ^§§^*p* < 0.01; ^§§§^*p* < 0.001; *p < 0.05; **p < 0.01; ***p < 0.001. § indicates 10058-F4 and cabozantinib (cabo) versus controls (ctr); * indicates 10058-F4 + Cabo versus respectively 10058-F4 and Cabozantinib.

Next, we selected three human HCC cell lines (JHH5, Hep3B, and Huh7) covering a range of MYC and MET levels to assess cell viability in response to their targeting. MYC was inhibited using 10058-F4, which interferes with MYC-MAX interaction and prevents transactivation of MYC target gene expression [46, 47]. Results showed that 10058-F4 reduced the viability of tested HCC cells in a dose-dependent manner (Fig. 6C). MET inhibition by cabozantinib, a multi-RTK inhibitor used in the clinic for HCC treatment, only partially interfered with the viability of human HCC cells we tested (Fig. 6C). Interestingly, the combination of both agents to block MYC and MET significantly reduced the viability of the human HCC cells we tested (Fig. 6C). We then used another MYC targeting agent, Omomyc, a peptide reported to act as a dominant negative agent blocking MYC function in cancer cells [48, 49]. After 72 h treatment, reduced viability was only observed on human Hep3B cells in the presence of Omomyc (Fig. 6D). Reassuringly, combined Omomyc and cabozantinib significantly reduced cell viability compared with single agents (Figs. 6D, S5B). The combined targeting displayed synergistic, or in a few cases additive, effects (Table S3). Biochemical experiments confirmed the effects of 10058-F4 and of Omomyc on MYC transcriptional function, as exemplified by the downregulation of SURVIVIN, MYC, and CYCLIN D1, although with variations between cell lines analysed and in relation to the MYC blocking agent used (Fig. 6E, F). No major changes were observed on MET levels (Fig. 6E, F), consistent with the maintained sensitivity of HCC cells to cabozantinib when used in combination with MYC blocking agents. We also assessed the effect of single and combined targeting of MYC and MET in HLE and HLF cells, which are classified as CL3 subclass. Cell viability assays corroborated the potency of combined MYC and MET targeting versus single treatments (Fig. 7A), and biochemical studies confirmed the downregulation of SURVIVIN, MYC, and CYCLIN D1 following MYC targeting (Fig. 7B).

**Fig. 8:**
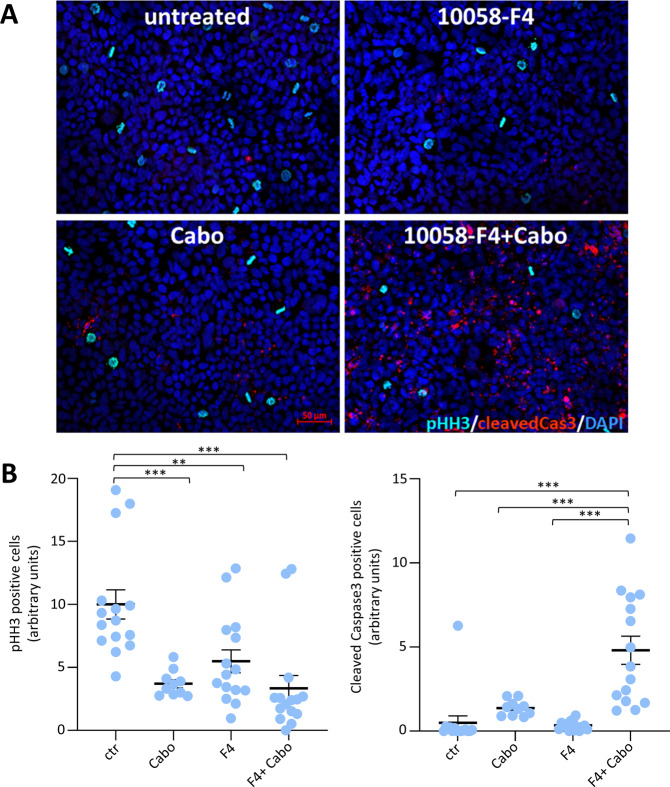
Combined blockage of MYC and MET converts a mild cytostatic effect (observed with individual targeting) into a robust cytotoxic effect. **A** Panels reports immunostaining with anti-phosphoHistoneH3 (pHH3; cyan) and anti-cleaved-Caspase3 (red) of Huh7 cells untreated (control), treated for 36 h with cabozantinib (3 µM; Cabo), 10058-F4 (60 µM), or 10058-F4 + Cabozantinib (10058-F4 + Cabo). Note a drastic increase of apoptotic cells in the presence of 10058-F4 + Cabozantinib. **B** Graphs reporting quantifications of anti-phosphoHistoneH3 (pHH3) and anti-cleaved-Caspase3 of Huh7 cells untreated versus single or combined treatments. A number of positive pHH3 cells was normalised over the number of cells. For pHH3, the mean of the controls was arbitrarily set to the value of 10. Cleaved-Caspase3 was quantified as the area of the red stain and has been normalised over the number of cells. The quantification was performed with Image J. Statistical analyses were performed by (one-way) ANOVA. **p < 0.01; ***p < 0.001.

To mechanistically explore the differences between single and combined treatments, we examined cell proliferation and apoptosis using anti-phospho-Histone-H3 and anti-cleaved-Caspase3 assays in Huh7 cells. We found that monotherapy with 10058-F4 or cabozantinib slightly reduced the proliferation rate of HCC cells, without triggering the expression of cleaved-Caspase3 (Fig. 8A, B). In contrast, combined treatment with 10058-F4 and cabozantinib induced apoptosis (Fig. 8A, B). Together, these results indicate that MYC targeting confers vulnerability of HCC cells to cabozantinib, converting a partial cytostatic (triggered by single treatment) into a cytotoxic effect (achieved by combined treatment).

## Discussion

In the present study, we report the generation of an “inside-out” mouse model that recapitulates the coexistence of *MYC*^*high*^*/MET*^*high*^ in a subset of HCC patients. Furthermore, we document functional cooperation of MYC and MET in HCC development from normal hepatocytes, even in the context of wild-type surrounding cells with no ongoing liver injury. Our findings illustrate how hydrodynamic tail vein injection combining two “open” predisposition genetic alterations, the *Alb-R26*^*Met*^ mice and *Myc*, can generate clinically relevant inside-out models of HCC subgroups. Additionally, the *MYC*^*high*^*/MET*^*high*^ model exemplifies the unique molecular traits and vulnerabilities that characterise combinations of genetic drivers.

By analysing five different databases, we showed that the percentage of *MYC*^*high*^*/MET*^*high*^ HCC patients varies among cohorts, ranging from 3 to 57%, possibly reflecting factors characterising the population of patients included in each cohort. We did not find any evident aetiology, risk factors, genetic aberrations, or molecular characteristics associated with the *MYC*^*high*^*/MET*^*high*^ subgroup. Thus, the coexistence of *MYC*^*high*^*/MET*^*high*^ in HCC patients is likely linked to factors that are not predominantly used to classify HCC patients and is part of the heterogeneity characterising this cancer. Nevertheless, it should be noted that the available clinical data corresponding to each patient in these HCC cohorts was limited or incomplete in most cases. Therefore, it is difficult at this stage to firmly conclude the absence of clinical factors that might characterize the *MYC*^*high*^*/MET*^*high*^ population. A rather intriguing aspect is that *MET* genetic mutations, while predominantly absent in adult patients, have been reported in paediatric HCC [17, 50]. Similarly, *MYC* amplification is an early event in HCC and has been associated with younger age of onset, and poorer prognosis [14, 51, 52]. Future studies using large paediatric HCC cohorts will clarify whether the *MYC*^*high*^*/MET*^*high*^ correlates with specific factors.

Our molecular studies comparing tumours from *Alb-R26*^*Met*^ mice versus those originated by hydrodynamic tail vein injection of *MYC* and *Cre* recombinase plasmids (to delete the stop cassette) in *R26*^*stopMet*^ mice indicate that MYC transcriptional activity may be capable of switching the molecular traits of HCC in a context of high MET levels. Whereas *Alb-R26*^*Met*^ tumours are *Afp*, *Spp1*, *Gpc3*, and *Epcam* positive, as we previously documented [29], *Myc-R26*^*Met*^ tumours do not express these markers (or at lower levels). Moreover, *Mki67* levels were about tenfolds increased in *Myc-R26*^*Met*^ compared with *Alb-R26*^*Met*^ tumours. Such a switch in expression markers associated with MYC resembles the expression of *Epcam* and *Krt19* in *Myc-HRas*^*G12V*^, whereas they are undetectable in *Myc-p53*^*shRNA*^ tumours [53]. This might indicate that the vast heterogeneity in marker expression observed in HCC is the outcome of the combinatorial action of distinct inputs. In the case of MYC and MET, it is tempting to speculate that MYC superimposes molecular traits over those otherwise present in MET tumours. Alternatively, sets of markers associated with MYC are refined by the signalling context in which MYC operates. The analysis we exemplified in Fig. 5D on a subset of markers in murine HCC tumours generated by the concomitant overexpression of MYC with different known oncogenes by hydrodynamic tail vein injection (GSE148379, [6]) rather supports this possibility. Indeed, the presence of MYC results in a highly heterogeneous rather than homogeneous signature. The possibility that MYC superimposes molecular traits according to the oncogenic context in which it operates could explain the distinct molecular characteristics in MYC plus MET tumorigenesis, not present in other models. These findings documenting a switch in genes expressions such as *Hmga1* and *Csp1* (Hep-Par1) could be informative for future studies exploring the identity of cells in the context of the inter- and intra-tumoral heterogeneity of HCC patients, of the dynamics in cell population changes during treatment, and particularly in relation to the emergence of resistant subpopulations. For these studies, single-cell RNA-seq will be particularly appropriate to determine the identity of distinct HCC cells in relation to *MYC* amplification/expression levels.

Intriguingly, our RT-qPCR analysis revealed that *Myc-R26*^*Met*^ tumours present a different expression pattern of several immune checkpoints as compared with *Alb-R26*^*Met*^ tumours. This suggests that MYC impacts the microenvironment, as we illustrate here in relation to specific immune signals. However, immunostaining showed that tumours are overall “cold” for CD3-positive T-cells. In humans, immunotherapy in HCC alone has a low response rate, and combination therapies can boost the response. It is tempting to speculate that MYC overexpression could remodel the HCC immune microenvironment, as proposed in other solid cancers [43, 44]. In view of these findings, it may be relevant to determine whether the *MYC*^*high*^*/MET*^*high*^ group of HCC patients could especially benefit from the use of combination therapies to boost the response rate to immunotherapy with anti-CTLA4 and/or anti-PD-L1 treatments.

As is the case with most of the transcription factors, the development of drugs that directly target MYC has been very challenging. Strategies for depleting MYC mainly rely on targeting its expression or its post-translational modifications. A dominant-negative form of MYC named Omomyc has been reported for its capability to penetrate in cancer cells, inhibit MYC transcriptional activity and function, and trigger tumour regression [48, 49]. Studies have shown that Omomyc is well-tolerated, leading only to mild, reversible side effects [48]. Based on these findings, Omomyc is currently in clinical development for the treatment of several cancer types. Our in vitro studies using a subset of human HCC cell lines showed a degree of vulnerability to combinatorial targeting of MET and MYC with cabozantinib and Omomyc or 10058-F4, respectively. This responsiveness is particularly relevant as the HCC cells we tested are otherwise only partially - or not responsive - to single drug treatment. This combinatorial treatment leads to a cytotoxic effect on HCC cells, not achieved using drugs individually, which only elicit a moderate cytostatic effect. It should be noted that the effectiveness of targeting MYC or both MYC + MET varies among cell types. For example, MYC blockage makes Hep3B and HLE cells more sensitive to cabozantinib than JHH5, Huh7, and HLF cells. No evident correlations were observed between sensitivity and expression levels of MYC and/or MET, indicating that vulnerability to MYC + MET blockage might be associated to other signalling characteristics. This configuration is similar to other single and combinatorial treatments for which the identification of signatures for selecting the most responding patients remains a major challenge. The concept of targeting MYC to confer responsiveness to MET inhibition is supported by previous studies showing that MYC blockage overcomes the resistance of other types of cancer cell lines to MET inhibitors [54]. Therefore, it is tempting to speculate that MYC blockage confers responsiveness of HCC cells to cabozantinib by reducing the expression levels of MYC targets, thus providing a higher degree of dependency on MET signalling support. Future studies will be instrumental in documenting how vulnerability to MYC blockage could be exploited to potentiate other RTK targeting agents already approved in HCC, including sorafenib, lenvatinib, and regorafenib. Moreover, it would be interesting to assess whether this combinatorial treatment could be a relevant approach to confer the vulnerability of HCC cells to other agents used in the clinic, particularly to immunotherapies.

## Materials and methods

### *Alb-R26*^*Met*^ mice

The generation of the *R26*^*stopMet*^ mice (international nomenclature *Gt(ROSA)26Sor*^*tm1(Actb-Met)Fmai*^) carrying a conditional mouse-human chimeric *Met* transgene inserted at the *Rosa26* locus has been previously reported [25, 26, 55]. In the *R26*^*stopMet*^ model, slightly enhanced wild-type MET levels are achieved following the removal of the stop cassette using the Cre recombinase [29]. In the *Alb-R26*^*Met*^ mice, with increased MET levels in the liver, tumours spontaneously form overtime, recapitulating the most aggressive HCC patient subtype defined as “proliferative-progenitor”, primary resistance to drugs used in the clinic, and the molecular heterogeneity of patients [26, 29]. The *Alb-R26*^*Met*^ HCC, as the “proliferative-progenitor” patient, is characterised by a striking enrichment in genes that are simultaneously overexpressed and hypermethylated in gene body CpG islands (CGIs) [31, 32]. The mouse line expressing Cre recombinase under the *Alb* promoter (*B6.Cg-Tg(Alb-cre)21Mgn/J*) was obtained from the Jackson Laboratory. *Alb-R26*^*Met*^ mice were generated by crossing the *R26*^*stopMet*^ and *Alb-Cre* mice [29, 31]. Mice were maintained in a 50% mixed 129S2/SvPasOrlRj and C57BL/6JRj background and genotyped by PCR analysis of genomic DNA as previously reported [25, 26]. Only male mice were used in these studies, at the age ranging from 10 and 20 weeks old.

### Hydrodynamic tail vein injection (HTVI)

For in vivo studies, we used the following plasmids: for *Cre* expression, AAV.TBG.PI.Cre.rBG (Addgene Plasmid #107787); for *Myc* expression, carrying as well the *Sleeping Beauty* transposase construct, we used a CAG promoter-driven MYC expression plasmid derived from pKT2/Fah-Myc//SBK [56], which we named pKT2/Myc//SBK. Control and *R26*^*stopMet*^ male mice were injected with 10 µg of AAV.TBG.PI.Cre.rBG plasmid to delete the stop cassette, leading to *Met* overexpression, with 10 µg of pKT2/Myc//SBK plasmid to overexpress *Myc*, or 10ug of each plasmid to overexpress both *Met* and *Myc*. Plasmids were injected in a final volume equivalent to 10% of the mouse weight (ml/mg) in Ringer’s lactate-buffered solution (Alfa Aesar, ThermoFisher, J67572). HTVI was performed in male mice at age ranging from 10 and 20 weeks old.

### Histology and immunohistochemistry

Liver tumours were dissected and processed for DNA, RNA, and protein analyses as described [31]. Mouse livers (four *Alb-R26*^*Met*^ tumours with adjacent livers from four mice and eight *MYC-R26*^*Met*^ tumours with adjacent livers from five mice) were embedded in OCT (Fisher), frozen, and cryosectioned. The slides were fixed using 4% paraformaldehyde, then used directly for immunofluorescence staining as previously reported [8]. Three non-overlapping images were taken (×20 objective) of each stained tumour and adjacent liver using Zeiss LSM 780 (Zeiss, Dublin, CA, USA) or Zeiss LSM 880 (Zeiss) laser-scanning confocal microscopes. The staining was scored semi-quantitatively as no staining (<2% of cells were immunoreactive), weak (either diffuse weak staining, or weak or strong focal staining in <30% of tumour cells), and strong (strong staining of ≥30% of tumour cells). To obtain the Ki67 index, three areas of highest nuclear labelling (‘hot spots’) were selected, and the percentage of positively stained tumour cells among the total number of tumour cells was calculated [57], using Fiji image processing software [58].

### Total mRNA extraction

Total mRNA from frozen tissues was isolated using the RNeasy Mini Kit (Qiagen), according to the manufacturer’s instructions. DNase (Qiagen) treatment was included to eliminate genomic DNA. mRNA was extracted from frozen samples after homogenising 20 mg of tissue in the RTL lysis buffer (Qiagen) supplemented with β-mercaptoethanol; samples were centrifugated at 6300 rpm twice for 30 s using Precellys 24 (Bertin technologies), then processed by using the RNeasy Mini Kit (Qiagen). The quality and concentration of RNA were evaluated with Nanodrop (ThermoFisher).

### cDNA and quantitative RT-PCR analysis

cDNA was synthesised using a Reverse Transcription Kit (iScript Supermix, Bio-Rad #1708841). PCR reactions were performed using SYBR GreenERqPCR SuperMix (ThermoFisher Scientific, #11761) and specific primers designed with PrimerBlast NCBI tool (1 µM; Table S1). Expression levels were quantified using the comparative Ct method (2^−ΔΔCT^ method) with the housekeeping genes *Gapdh* as a control for internal normalisation, and results are expressed as RQ = 2^^(−ΔΔCT)^.

### Cell culture

HepG2 (ATCC HB-8065) cells were grown in EMEM (ThermoFisher Scientific). HLF (JCRB0405), Huh7 (JCRB0403), and Hep3B (ATCC HB-8065) cells were grown in DMEM (ThermoFisher Scientific). HLE (JCRB0404) and SNU449 (ATCC CRL-2234) cells were grown in RPMI (Gibco), while JHH5 cells were grown in William E medium (Gibco). Unless differently indicated, all the media were supplemented with 10% foetal bovine serum and penicillin-streptomycin. JHH5, SNU449, and Huh7 cells were kindly provided by S. Rebouissou. All other cells were obtained from ATCC or JCRB without further authentication. Cells were cultured in an incubator at 37 °C and 5% CO_2_. All cells were tested by PCR-based assay to verify that they were free of *Mycoplasma* contamination.

### Drug treatment and cell viability assay

The drugs used on cell cultures were: cabozantinib (3 and 5 µM; TargetMol), the small molecule MYC inhibitor 10058-F4 (20, 40, and 60 µM; TargetMol), and Omomyc (10 and 30 µM; kindly provided by L. Soucek and J. Whitfield, Vall d’Hebron Instituto de Oncología and Peptomyc) [48], currently in phase 1/2 clinical trial for other solid cancers (ClinicalTrials.gov Identifier: NCT04808362). Cell viability assays were performed as previously reported [29]. Briefly, cells were seeded in 150 µL volume of medium per well in 96 well plates (10,000 or 3000 cells/well for 48 h or 72 h treatment, respectively) in the presence of 10 % serum. After 24 h, inhibitors were applied, either individually or in combination, at the indicated concentrations. After 48 h and 72 h, cell viability was assessed with the Cell Counting Kit-8 colourimetric assay (TargetMol). Colourimetric signals were measured with a luminometer microplate reader (Berthold). Cell viability was normalised to non-treated (NT) cells. Data obtained from viability assays are the mean of three to six independent experiments performed in triplicate. To classify the effects of combined treatment in synergistic, additive, or antagonistic, the Bliss independence method was applied [59].

### Western blots

Protein expression levels in HCC cell lysates of non-treated and treated cells (Omomyc and 10058-F4 for 24 h) were analysed by western blot, using the EBM protein extraction buffer (20 mM Tris-HCl pH 7.5, 150 mM NaCl, 5 mM EGTA, 5 mM EDTA, 10% glycerol, 1% Triton) supplemented by a cocktail of protease (5 µg/ml leupeptin, 5 µg/ml pepstatin, 2 µg/ml aprotinin, 5 mM PMSF) and phosphatase (10 mM NaF, 1 mM NaPP, 1 mM Na_3_VaO_4_, 10 mM β-glycerophosphate) inhibitors, following previously described procedures [26, 60]. Quantification of western blots was done using FIJI software. Full blots of gels in which the acquisition of ECL signal performed using the BioRad imager system was merged with a picture of the membranes, and the corresponding Ponceau red stain is reported in Figs. S6 and S7. The antibodies used for western blots are reported in Table S2.

### Immunocytochemical analyses on cultured cells

Human HCC cell lines were fixed and processed for immunofluorescent staining, as previously reported [27, 28, 32]. Briefly, after 36 h treatment with 10058-F4 and cabozantinib, alone or in combination, Huh7 cells were fixed in 4% paraformaldehyde (PFA) for 10 min and then washed three times with PBS for 5–10 min. The fixed samples were then permeabilized with 0,3% TritonX-100, blocked with 3% BSA, 2% Donkey Serum, and 0.3% TritonX-100 in PBS for 1 h. The blocking solution was used to dilute the primary antibodies and the samples were incubated overnight at 4 °C. The day after, cells were washed with 0.3% TritoX-100 and then incubated with secondary antibodies (diluted 1:500 + DAPI 5 µg/ml) for 1 h at room temperature. Coverslips were then mounted using ProLong^™^ Gold Antifade Mountant (Thermo Fisher Scientific, ref: P10144), and images were taken with a Zeiss AxioImager APO Z1 microscope.

### Analysis of publicly available human RNA-seq data

The human RNA-seq data from LIHC-TCGA was available through the Firebrowse portal, and both LICA-FR and LIRI-JP through ICGC Data Portal (https://dcc.icgc.org/). The data from GSE62232 and GSE138485 cohorts were downloaded from the NCBI GEO data portal (https://www.ncbi.nlm.nih.gov/gds). The calculation of Log_2_ Fold Change (log_2_ tumour sample/control sample) was applied to each individual patient using as a control the mean of control samples when available (LIRI-JP, TCGA, GSE62232 and GSE138485 cohorts). To stratify the HCC patients into four subgroups (*MYC*^*high*^*/MET*^*high*^, *MYC*^*low*^*/MET*^*high*^, *MYC*^*high*^*/MET*^*low*^, and *MYC*^*low*^*/MET*^*low*^), we considered high expression when Log_2_FC > 0 and low expression when Log_2_FC < 0. For the LICA-FR cohort, as no normal samples are publicly available, we used instead the data available in the GTEX portal (https://gtexportal.org/home/) for healthy liver samples to perform the Log_2_FC and stratify the HCC patients into the four groups above mentioned.

### Statistical analysis

All data were analysed using GraphPad Prism software (version 7 and version 8). Statistically significant differences were estimated by applying an unpaired Student t-test to data showing normal distribution (results are expressed as the mean ± standard error of the mean; SEM), one-way ANOVA (for viability assays) and two-way ANOVA (for RT-qPCR analysis) or Mann–Whitney test in all other situations (results are expressed as dots; each dot corresponds to each analysed sample). All statistical tests were two-sided. Statistical significance (*p*-values) was defined as not significant (ns):*p* > 0.05; * or ^§^*p* < 0.05; ** or ^§§^*p* < 0.01; *** or ^§§§^*p* < 0.001. Significance is indicated in the Figures. Only significant differences were indicated with the asterisk in panels.

## Supplementary information

Supplementary Figure and Table Legends

Supplementary Figures S1-S5

Supplementary Table S1

Supplementary Table S2

Supplementary Table S3

Supplementary Figures S6-S8 - Full and uncropped western blots

Reproducibility checklist

## Data Availability

All data was downloaded from publicly available databases for which the links are provided in the Material and methods section.
