# Supplementary material for: MYC and MET cooperatively drive hepatocellular carcinoma with distinct molecular traits and vulnerabilities
Source: Cell Death Dis. 2022 Nov 24;13(11):994. doi: 10.1038/s41419-022-05411-6 (PMC9700715; doi:10.1038/s41419-022-05411-6)
Supplement: Supplementary file 1 — Supplementary Figure and Table Legends [file 41419_2022_5411_MOESM1_ESM.docx]

**MYC and MET cooperatively drive hepatocellular carcinoma with distinct molecular traits and vulnerabilities**

Celia Sequera^1,#^, Margherita Grattarola^1,2,#^, Agnes Holczbauer^3^, Rosanna Dono^1^, Stefania Pizzimenti^2^, Giuseppina Barrera^2^, Kirk Wangensteen^3,*^, and Flavio Maina^1,*^

**SUPPLEMENTARY MATERIAL 1**

**Figure Legends**

**Fig. S1. High expression levels of *MYC* and *MET* co-occur in a subset of HCC patients. (A)** Heatmap reporting LICA-FR cohort with HCC patients organized according to the expression levels of *MYC* and *MET*, and subdivided into four subgroups: *MYC^high^/MET^high^*, *MYC^low^/MET^high^*, *MYC^high^/MET^low^*, and *MYC^low^/MET^low^*. The percentage of patients in each subgroup is indicated on the left of each heatmap. As the LICA-FR cohort has no paired normal tissue to the tumour samples, the fold changes were calculating taking the GTEX normal liver samples.

**Fig. S2. Hepatocellular characteristics of *Myc-R26^Met^* and *Alb-R26^Met^* tumours. (A-F)** Graphs reporting the mRNA expression levels by RT-qPCR of HCC markers related to: MYC targets (**A**: *Eif4e*), HCC characterization (**B**: *Saa1, Fabp1*), progenitor cells (**C**: *Hnf4a, Krt19*), Wnt pathway (**D**: *Glul, Lgr5, Oat*), metabolism (**E**: *Ark1b10, Gpx2*), and HCC differentiated markers (**F**: *Arg1*, *Ctlc*, *Hsp1*, *Yap1*) in *Myc-R26^Met^* versus *Alb-R26^Met^* tumours. Values were normalized with the *GAPDH* housekeeping gene, and expressed as RQ, all values relative to *Alb-R26^Met^* tumours. Statistical analyses were performed by Mann-Whitney. No significant changes were found between the two tumour types, except for *Ctlc* (*: p≤0.05;).

**Fig. S3. Immune-related characteristics of *Myc-R26^Met^* and *Alb-R26^Met^* tumours. (A-B)** Graphs reporting the mRNA expression levels by RT-qPCR of immune checkpoints (*TIM-3*, *Galectin-9*, *PD-L1*) in *Myc-R26^Met^* versus *Alb-R26^Met^* tumours. Values were normalized with the *GAPDH* housekeeping gene, and expressed as RQ, all values relative to *Alb-R26^Met^* tumours. Statistical analyses were performed by Mann-Whitney. No significant changes were found between the two tumour types. **(C)** HIS staining using anti-CD3 (green), anti-MET (red), and DAPI (blue).

**Fig. S4. *MYC* and *MET* expression levels and correlation in a panel of human HCC cell lines. (A)** Data were extracted from <https://lccl.zucmanlab.com/hcc/molecularFeatures/rnaExpression?index=1>. **(B-C)** Graphs reporting correlation in the expression levels of *MYC* and *MET* in the three human HCC cell line clusters. No significant correlation was observed, except in CL3 cluster (p=0.0399).

**Fig. S5. (A)** Graphs reporting quantifications of expression of the indicated proteins, based on densitometric analysis by Image J. Measures were normalized using ACTIN or TUBULIN; the quantification was done setting as 1 the expression of the cell line with the lowest amount of protein. *: P<0.05; **: P<0.01; ***: P<0.001. **(B)** Graphs reporting the viability of cells untreated or in the presence of Omomyc (10, 30µM) either alone or combined with Cabozantinib (5µM) for 48 h in indicated human HCC cell lines. Note an intermediate response compared to cells treated for 72 h (reported in Fig. 6D), reflecting the downregulation of MYC targets after 24 h treatment with Omomyc. Reduced cell viability in the presence of Omomyc to: 88.73 % and 70.48 % in Hep3B with 10 and 30µM (p<0.01 and p<0.0001, respectively); of cabozantinib to: 59.54 % in JHH5 (p<0.0001), 72.57 % in Hep3B (p<0.0001), and 84.16 % in Huh7 (p>0.05); of Omomyc plus cabozantinib to: 30.84 % and 28.54 % in JHH5 with 10 and 30µM (p≤0.0001); 55.18 % and 33.27 % in Hep3B with 10 and 30µM (p≤0.0001); 83.92% and 61.41% in Huh7 with 10 and 30µM (p>0.05 and p<0.05, respectively).

**SUPPLEMENTARY TABLE LEGENDS**

**Table S1. Oligonucleotide primers for RT-qPCR analyses.**

**Table S2. List of antibodies used for western blot and immunofluorescence.**

**Table S3. Bliss independence method and values to determine synergistic versus additive effects.**

**SUPPLEMENTARY MATERIAL 2**

**Figure Legends**

**Fig. S6. Full unedited gels for data reported in Fig. 6A.** Full blots and Ponceau red stain are reported with the corresponding molecular weights. Red squares are used to highlight images reported in Fig. 6A.

**Fig. S7. Full unedited gels for data reported in Fig. 6E and F.** Full blots and Ponceau red stain are reported with the corresponding molecular weights. Red squares are used to highlight images reported in Fig. 6E-F.

**Fig. S8. Full unedited gels for data reported in Figure 7B.** Full blots and Ponceau red stain are reported with the corresponding molecular weights. Red squares are used to highlight images reported in Fig. 7B.
