# Supplementary material for: MYC and MET cooperatively drive hepatocellular carcinoma with distinct molecular traits and vulnerabilities
Source: Cell Death Dis. 2022 Nov 24;13(11):994. doi: 10.1038/s41419-022-05411-6 (PMC9700715; doi:10.1038/s41419-022-05411-6)
Supplement: Supplementary file 2 — Supplementary Figures S1-S5 [file 41419_2022_5411_MOESM2_ESM.pdf]

A

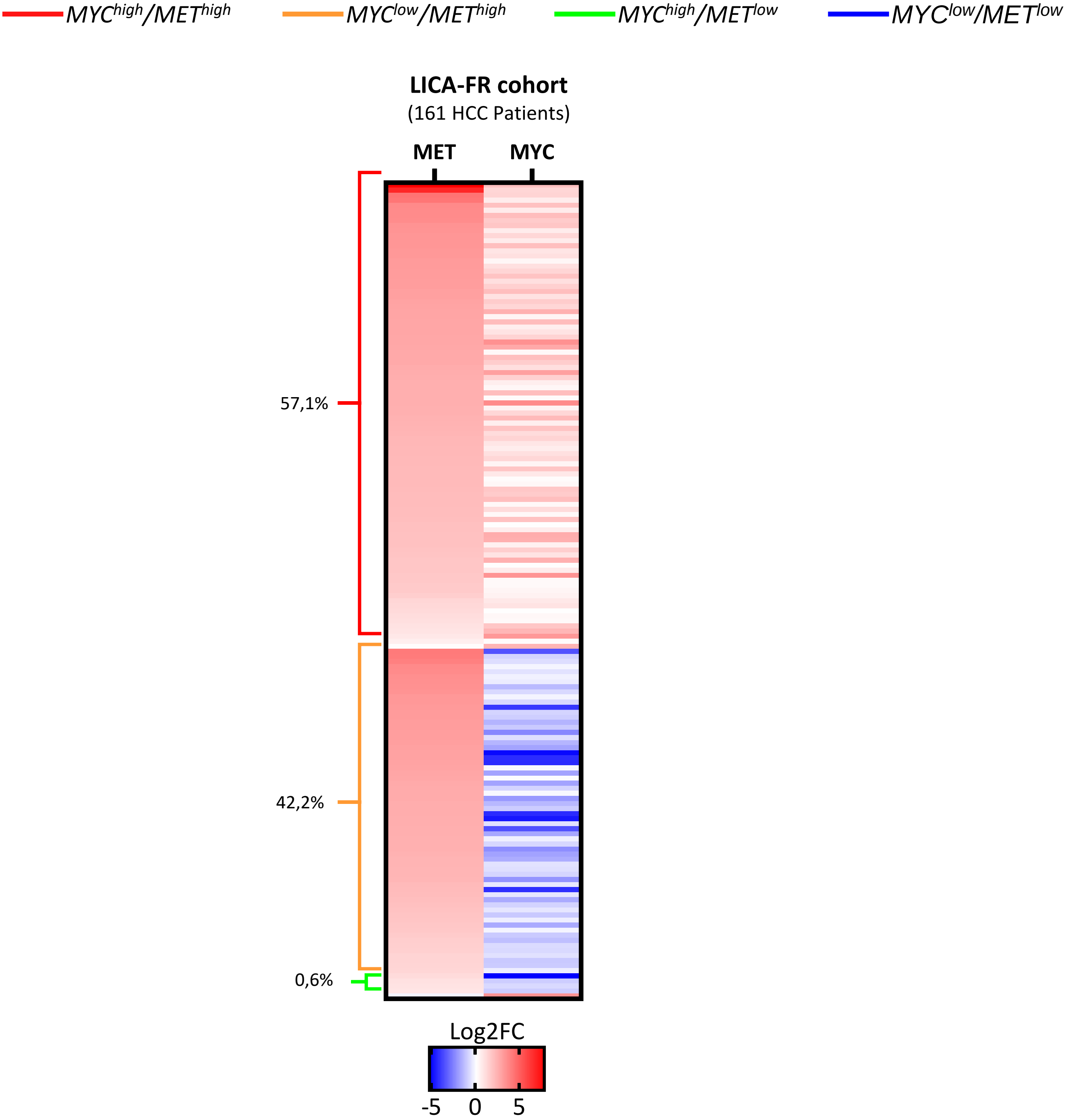

MYC^high/MET^high
MYC^low/MET^high
MYC^high/MET^low
MYC^low/MET^low
LICA-FR cohort
(161 HCC Patients)
MET
MYC
57,1%
42,2%
0,6%
Log2FC
-5
0
5

Figure S1

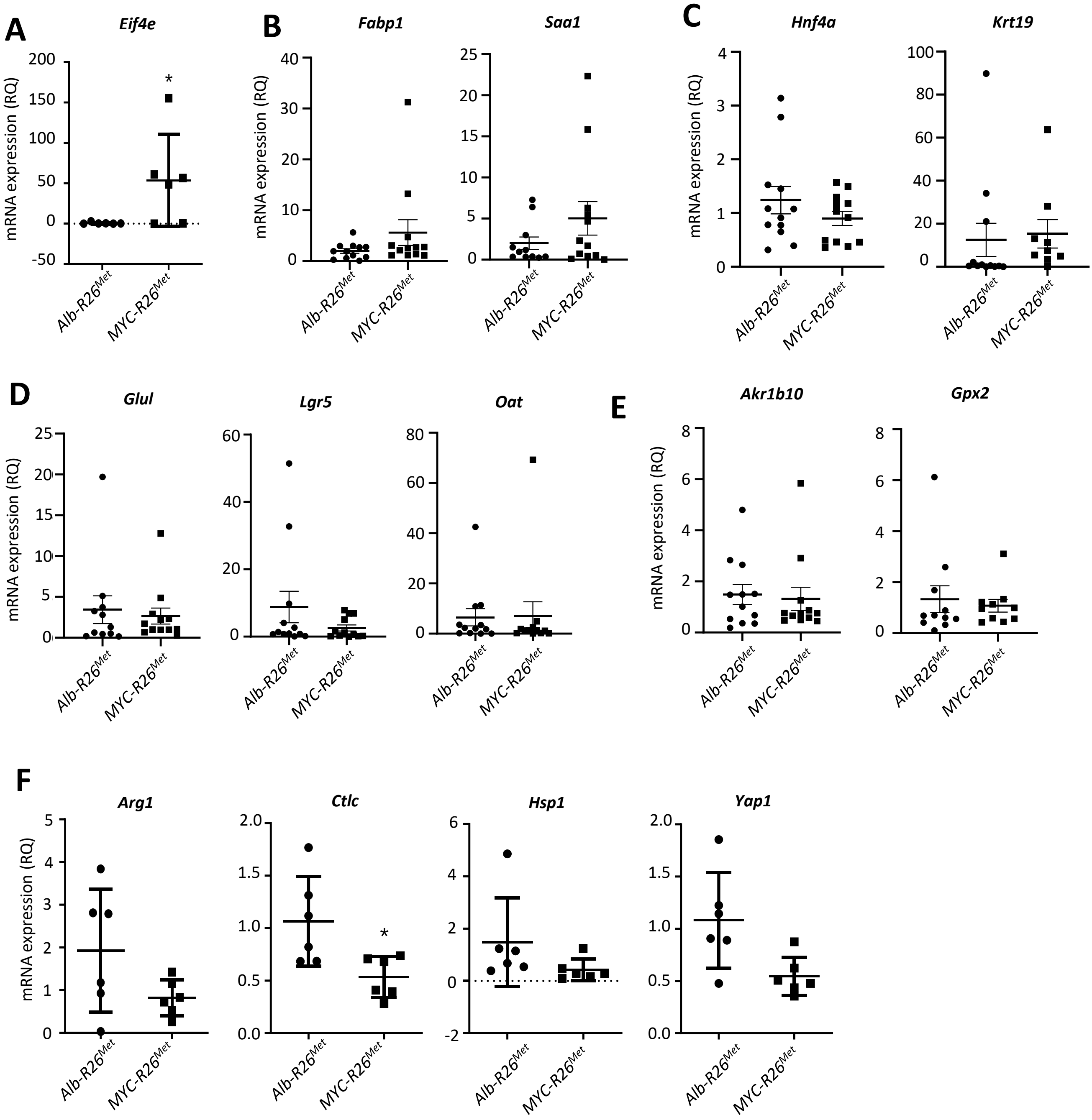
A
Eif4e
B
Fabp1
Saa1
C
Hnf4a
Krt19
D
Glul
Lgr5
Oat
E
Akr1b10
Gpx2
F
Arg1
Ctlc
Hsp1
Yap1
mRNA expression (RQ)
Alb-R26Met
MYC-R26Met
*

Figure S2

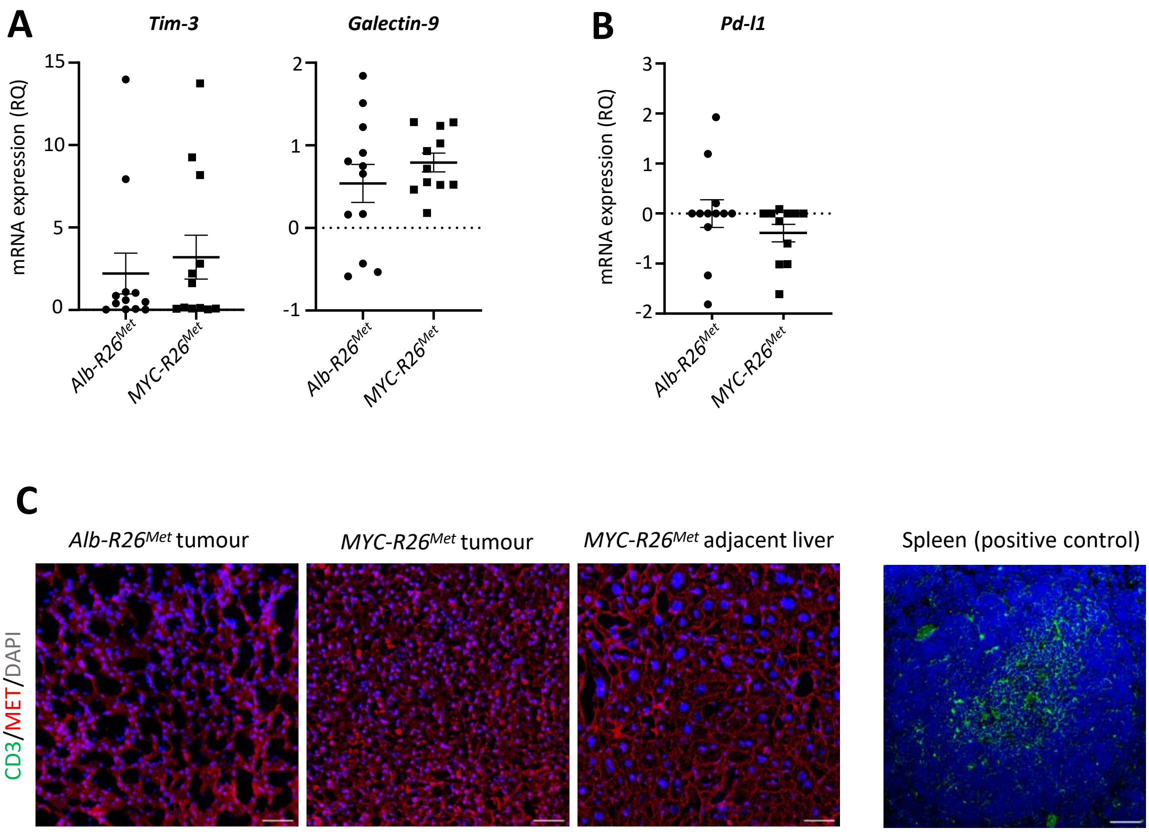
A
Tim-3
Galectin-9
mRNA expression (RQ)
15
10
5
0
2
1
0
-1
Alb-R26Met
MYC-R26Met
B
Pd-l1
3
2
1
0
-1
-2
C
Alb-R26Met tumour
MYC-R26Met tumour
MYC-R26Met adjacent liver
Spleen (positive control)
CD3/MET/DAPI

Figure S3

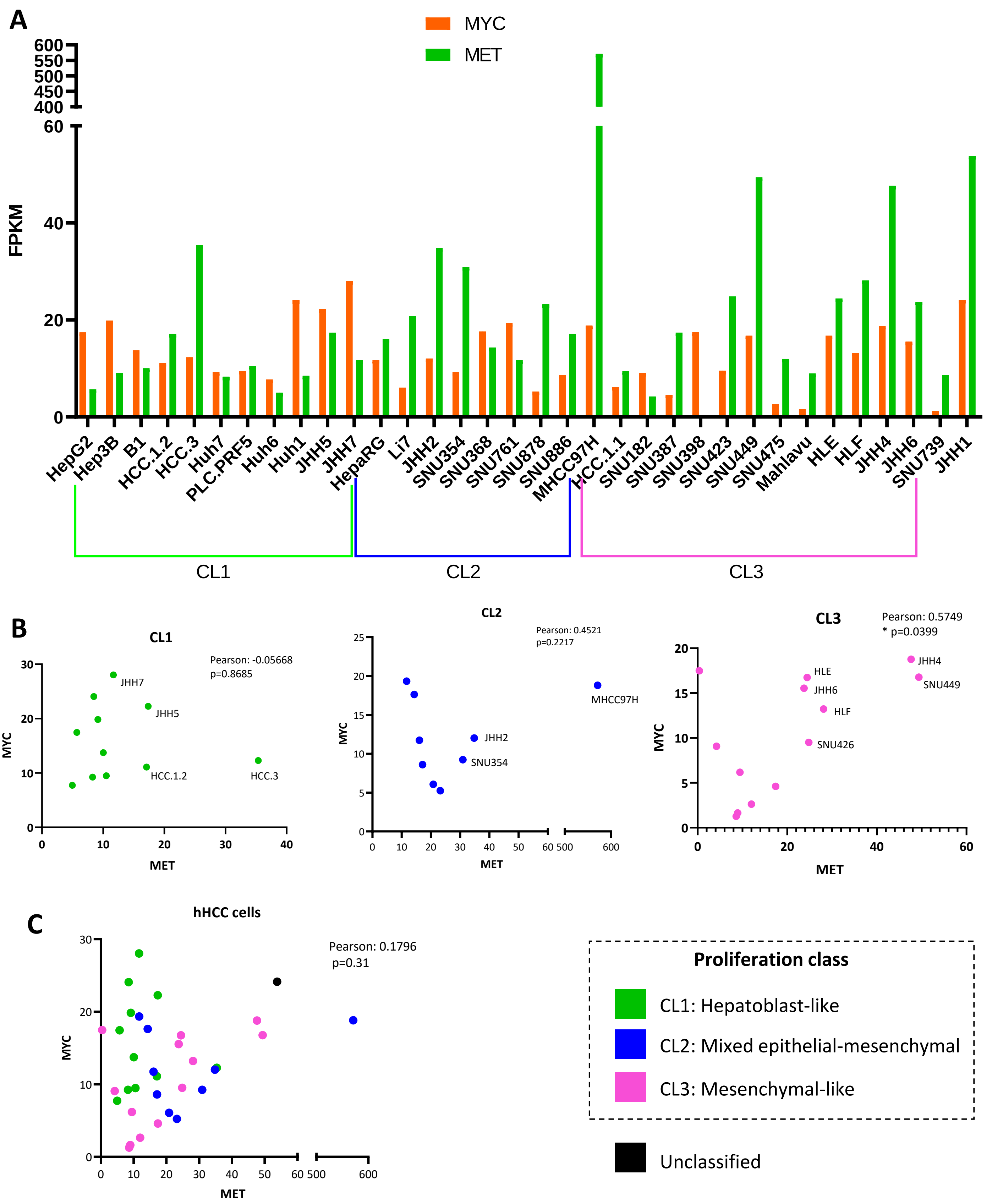
A
MYC
MET
FPKM
HepG2
Hep3B
B1
HCC.1.2
HCC.3
Huh7
PLC.PRF5
Huh6
Huh1
JHH5
JHH7
HepaRG
Li7
JHH2
SNU354
SNU368
SNU761
SNU878
SNU886
MHCC97H
HCC.1.1
SNU182
SNU387
SNU398
SNU423
SNU449
SNU475
Mahlavu
HLE
HLF
JHH4
JHH6
SNU739
JHH1
CL1
CL2
CL3
B
CL1
Pearson: -0.05668
p=0.8685
JHH7
JHH5
HCC.1.2
HCC.3
MYC
MET
CL2
Pearson: 0.4521
p=0.2217
MHCC97H
JHH2
SNU354
MYC
MET
CL3
Pearson: 0.5749
* p=0.0399
JHH4
SNU449
HLE
JHH6
HLF
SNU426
MYC
MET
C
hHCC cells
Pearson: 0.1796
p=0.31
MYC
MET
Proliferation class
CL1: Hepatoblast-like
CL2: Mixed epithelial-mesenchymal
CL3: Mesenchymal-like
Unclassified

Figure S4

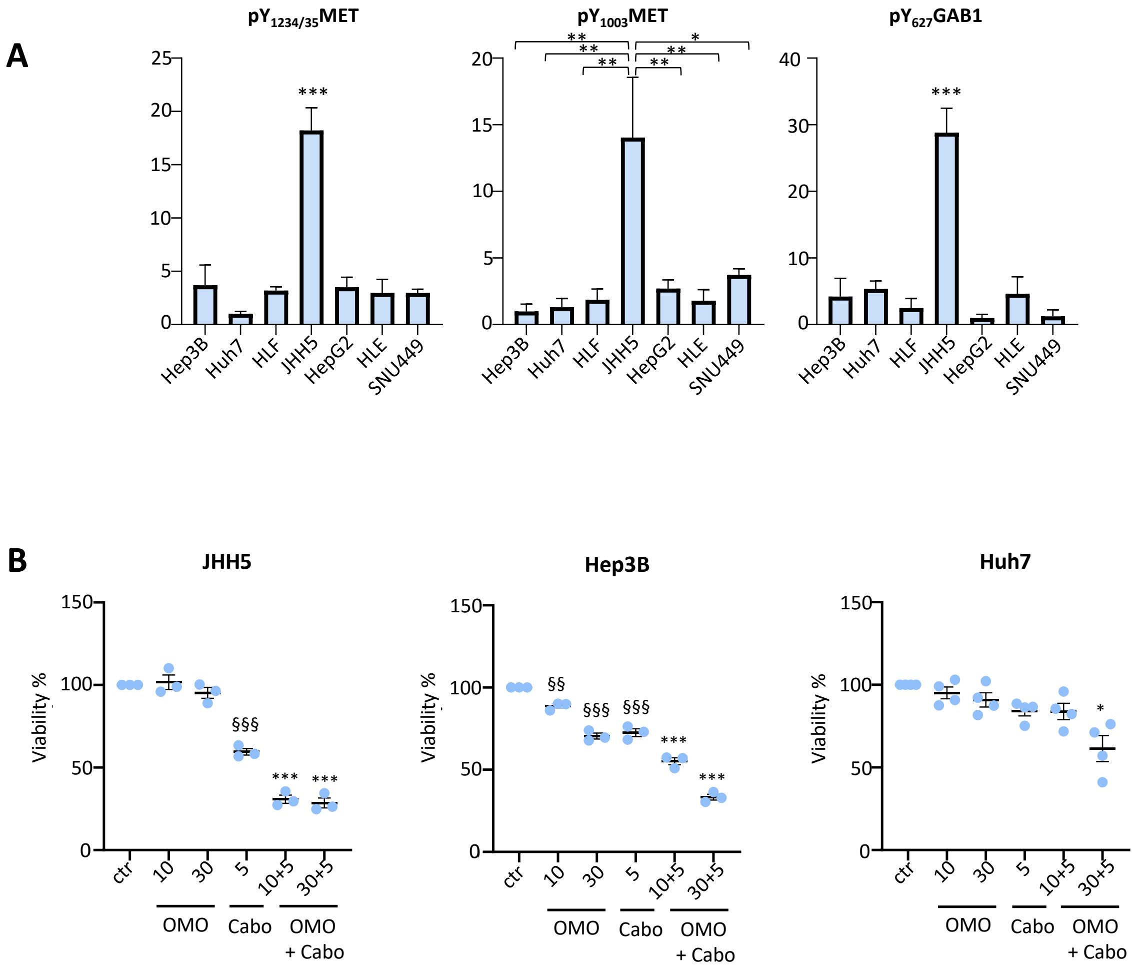
A
pY1234/35MET
pY1003MET
pY627GAB1
Hep3B
Huh7
HLF
JHH5
HepG2
HLE
SNU449
B
JHH5
Hep3B
Huh7
Viability %
ctr
OMO
Cabo
OMO + Cabo

Figure S5
