# Supplementary figures and images for: MYC and MET cooperatively drive hepatocellular carcinoma with distinct molecular traits and vulnerabilities
Source: Cell Death Dis. 2022 Nov 24;13(11):994. doi: 10.1038/s41419-022-05411-6 (PMC9700715; doi:10.1038/s41419-022-05411-6)

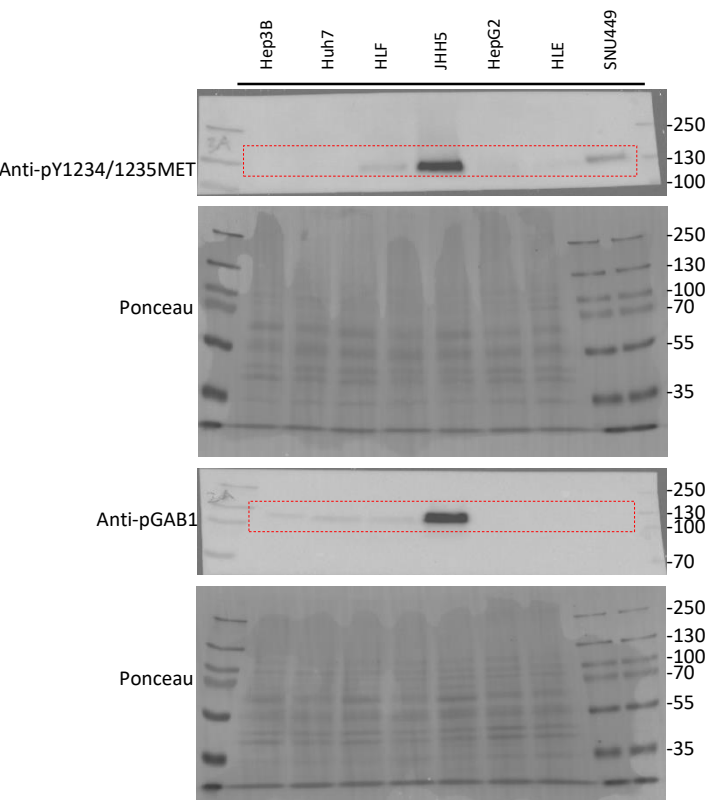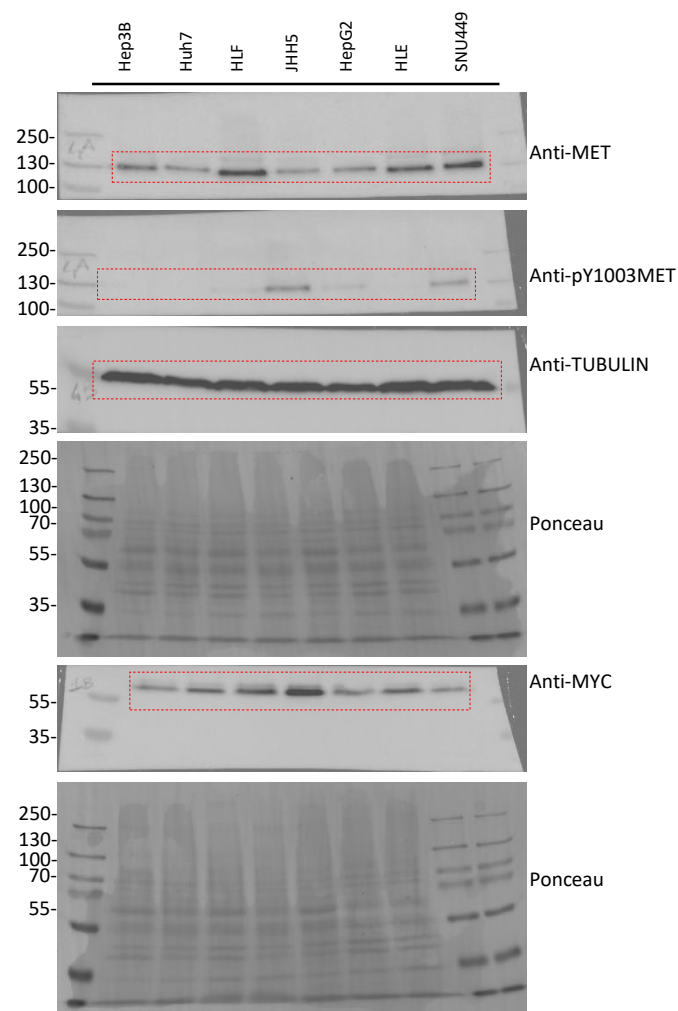

Figure S6

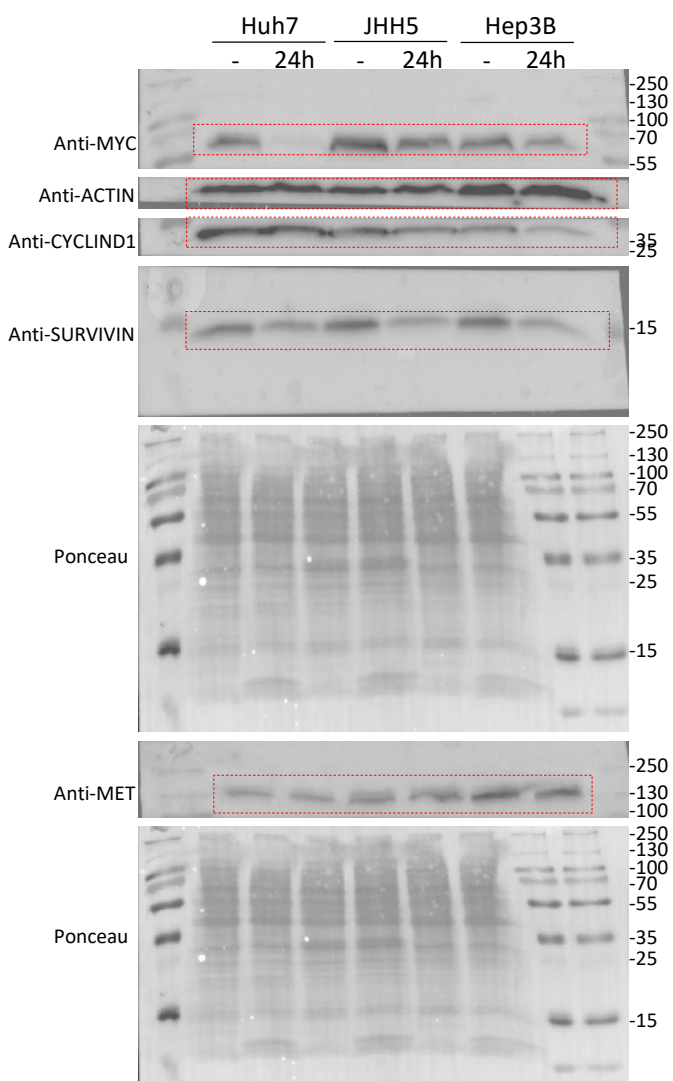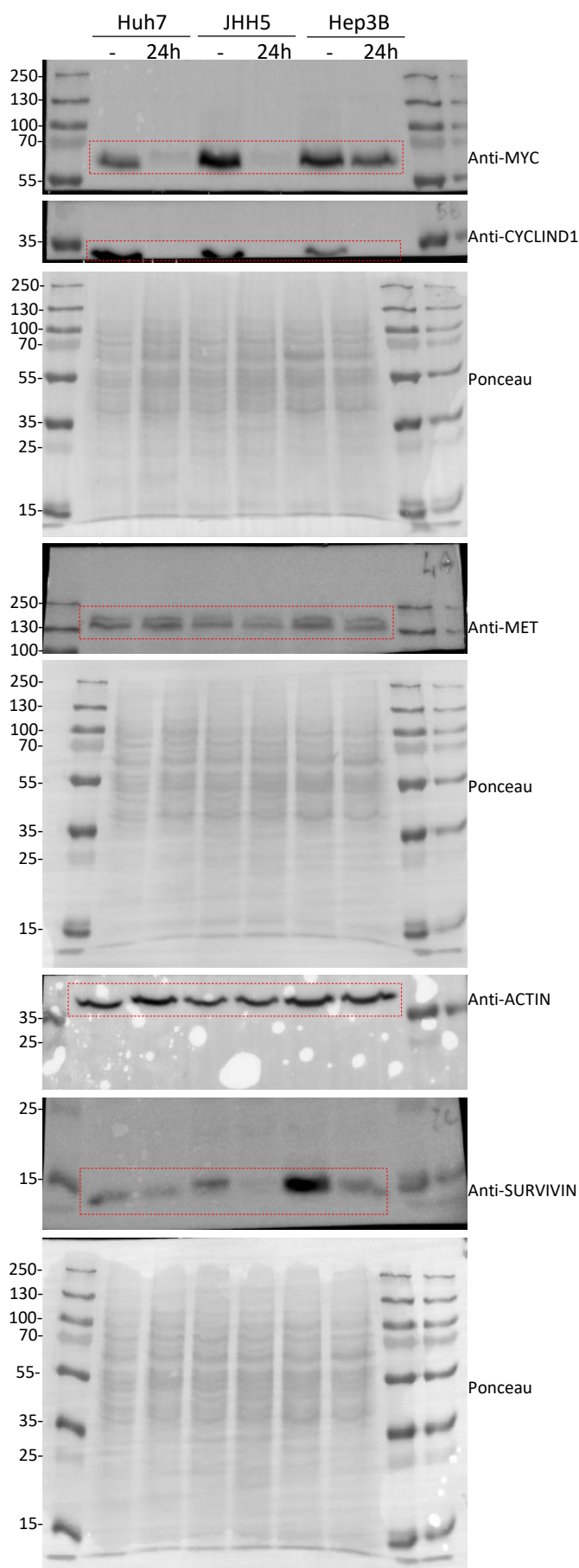

Figure S7

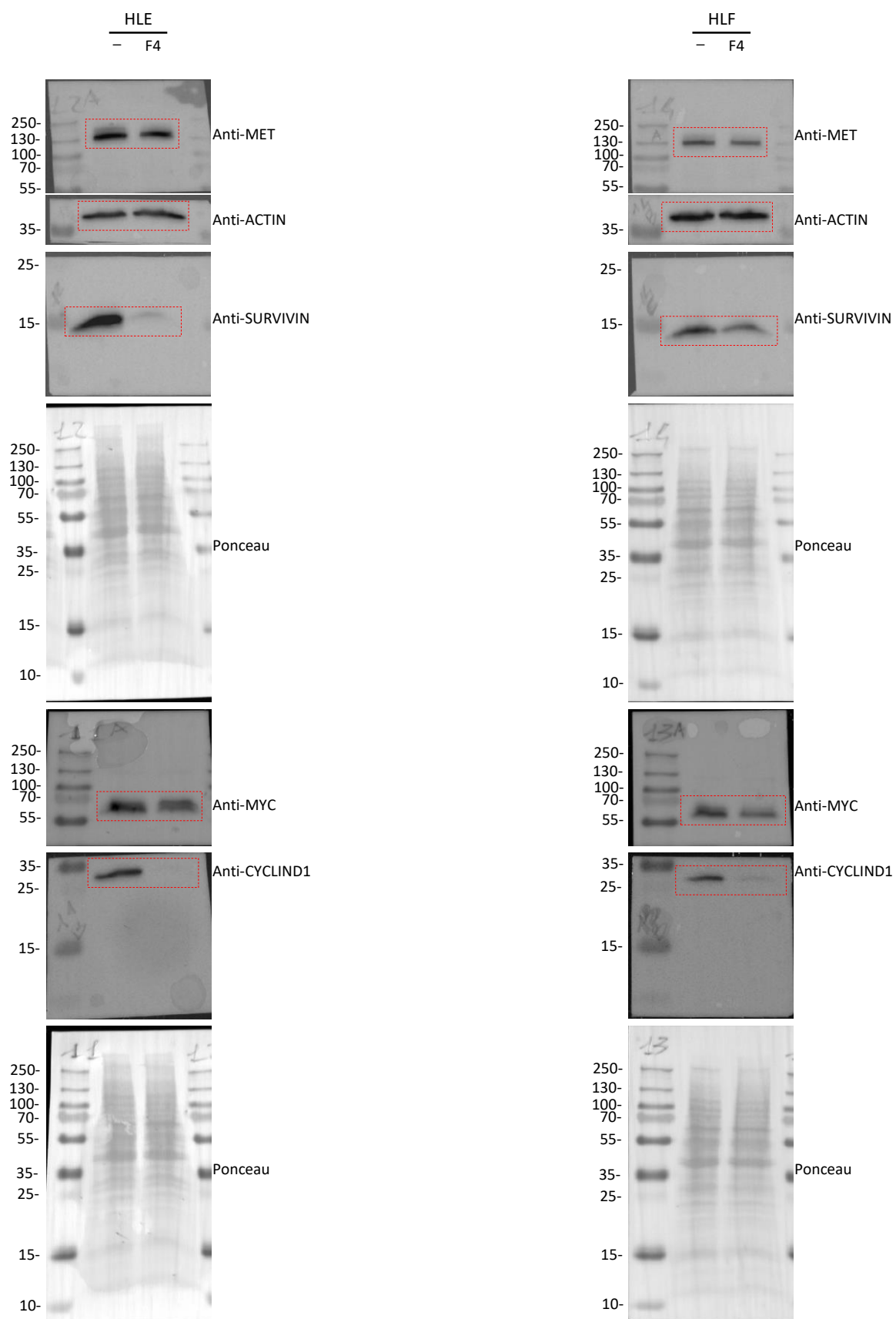

Figure S8

Supplement: Supplementary file 6 — Supplementary Figures S6-S8 - Full and uncropped western blots [file 41419_2022_5411_MOESM6_ESM.pdf]
